# Middle Meningeal Artery Embolization Using Non-Adhesive Liquid Embolic Agents for Chronic Resistant Migraine: Interim Feasibility and Safety Analysis of a Prospective Single-Arm Trial

**DOI:** 10.3390/jcm15208151

**Published:** 2026-10-20

**Authors:** Andrey Petrov, Ruslan Sharshebaev, Gulmira Biialieva, Sergei Ermakov, Arkady Ivanov, Ekaterina Samorukova, Anna Petrova, Julia Suvorova, Stalbek Akhunbaev

**Affiliations:** 1 North-Western District Scientific and Clinical Center Named after L.G. Sokolov, Federal Medical and Biological Agency, 194291 Saint Petersburg, Russia; s.v.yermakov@yandex.ru (S.E.); doctorsamorukova@yandex.ru (E.S.); petrovaanna2803@gmail.com (A.P.); juliavsuvorova@mail.ru (J.S.); 2 «Vedanta» Medical Center, International School of Medicine, International University of Kyrgyzstan, Bishkek 720054, Kyrgyzstan; ruslansharshebaev@gmail.com (R.S.); biyalievagulmira@gmail.com (G.B.); stal.ahunbaev@gmail.com (S.A.)

**Keywords:** chronic migraine, resistant migraine, middle meningeal artery, embolization, non-adhesive liquid embolic agent, ethylene vinyl alcohol copolymer, dura mater, trigeminovascular system, feasibility study

## Abstract

**Background/Objectives**: Chronic resistant migraine responds poorly to multiple classes of preventive therapy. The middle meningeal artery (MMA), along which nociceptive trigeminal fibers run, has been proposed as an interventional target. We report an interim feasibility and safety analysis of the first 10 patients in an ongoing prospective trial of MMA embolization with non-adhesive liquid embolic agents. **Methods**: Single-arm, open-label, multicenter, investigator-initiated trial at two centers (ClinicalTrials.gov NCT06848166; registered retrospectively). Of 318 patients assessed for eligibility, 16 (5.0%) met European Headache Federation criteria for resistant migraine and 10 were treated between October 2024 and November 2025; data cut-off was 5 September 2026. This interim analysis was not prespecified. Its main outcome, defined post hoc, was technical success—grade III or higher on the investigator-developed Saint Petersburg Arterial Dural Embolization Scale (SPADES)—reported per branch on an intention-to-treat basis and per patient (all four target branches); the registered primary efficacy endpoint, change in monthly migraine days at 12 weeks, is reported descriptively. **Results**: Of forty target branches, thirty were catheterized directly, six were filled retrogradely, and four were not embolized. Grade III or higher was achieved in 29 of 40 branches (72.5%; 95% CI 56.1–85.4) and in all four branches in four of ten patients (40%). The level reached by the catheter was related to branch tortuosity, and catheterizability of the parietal branch to the Adachi configuration. Anastomoses with the ophthalmic artery were present in 14 of 19 assessable arteries (73.7%). The embolic agent extended into the dural venous drainage in eight of ten patients without entering a sinus lumen. No serious procedure-related adverse event occurred (zero of ten; 95% CI 0–30.8); post-embolization headache lasting a median of 15 days occurred in nine patients, and all patients remained at modified Rankin Scale 0. Median monthly migraine days fell from 20.5 (IQR 18.0–22.5) at baseline to 8.0 (IQR 0.2–17.0) at 12 weeks, with a reduction of at least 50% in six of ten patients (60%; 95% CI 26–88); acute medication days fell in parallel. **Conclusions**: Bilateral MMA embolization with non-adhesive liquid embolic agents was technically feasible in this small series, which demonstrates initial procedural feasibility but cannot establish safety or efficacy. The clinical changes are uncontrolled and unblinded, cannot be separated from a placebo response, regression to the mean or reduced medication use, and require a sham-controlled trial.

## 1. Introduction

Chronic resistant migraine (CRM) is characterized by a lack of response to drug therapy with multiple lines of medications [1,2] and is associated with significant impairment of quality of life, high levels of disability [3], and comorbid anxiety and depression [3,4]. The pathophysiology of migraines is based on activation of the trigeminovascular system (TVS) [5], with the key component being the nociceptive fibers of the trigeminal nerve, which are concentrated along the branches of the middle meningeal artery (MMA), the main source of blood supply for the supratentorial dura [2,6,7]. Vasoactive peptides released from trigeminal nerve fibres activate CGRP (calcitonin gene-related peptide) and PACAP (pituitary adenylate cyclase-activating polypeptide) receptors in the MMA vascular wall [8]. This was long thought to cause artery dilatation [2] and thereby trigger a migraine attack. However, magnetic resonance angiography during provoked and spontaneous migraine attacks has shown that dilatation of the meningeal and extracranial arteries is not a prerequisite for migraine pain [9,10], and current concepts of the trigeminovascular system emphasize activation and sensitization of perivascular trigeminal nociceptors rather than vasodilatation per se [5,8,11]. Accordingly, we regard the MMA as a target because it carries the nociceptive trigeminal afferents, not because embolization prevents vasodilatation. MMA embolization with non-adhesive liquid embolic agents (NLEA) is effective for chronic subdural hematomas [12,13,14,15], and notably, many such patients reported headache improvement afterward [16].

Early attempts to treat migraine by targeting the MMA—intra-arterial infusion of pharmacological agents [17,18,19], coil embolization [20], and, most recently, bilateral occlusion with cyanoacrylate glue in a 10-patient series [21]—have been reported to be safe and potentially effective. The role of NLEAs for the treatment of CRM remains largely unknown. No standard approaches exist for selecting an embolic agent or occlusion level, and complicating factors such as proximal arterial anastomoses remain underexplored. The aim of this report is to describe our initial technical experience and to assess the feasibility and safety of bilateral MMA embolization using NLEA in patients with verified CRM; given the early phase and small cohort, clinical outcomes are reported as preliminary rather than confirmatory. To our knowledge this is the first report of MMA embolization with non-adhesive liquid embolic agents in chronic resistant migraine. A prospective self-controlled cohort of 15 patients treated with coils has recently been published and the feasibility and safety of MMA embolization with SQUID in a different indication were established in the STEM trial; the contribution of the present work is therefore the indication and embolic agent rather than the technique itself.

## 2. Materials and Methods

### 2.1. Study Design and Cohort

This is an interim analysis of the first 10 consecutive patients treated in an ongoing prospective, single-arm, open-label, multicenter, investigator-initiated study of middle meningeal artery (MMA) embolization with the non-adhesive liquid embolic agents SQUID 12 and SQUID 18 (Balt, Montmorency, France) in chronic resistant migraine (the **CHRO**nic **M**igraine **E**mbolization [CHROME] study; ClinicalTrials.gov identifier NCT06848166) [22]. The study is conducted at two centers in Kyrgyzstan and Russia.

The CHROME study is a pilot exploratory trial designed to generate preliminary estimates of the efficacy and safety of MMA embolization in this population and to inform the design of a future confirmatory trial. Patients are not randomized and are not allocated to groups; all enrolled patients undergo embolization, and the study is open-label, without masking of patients, operators or outcome assessors. Planned enrolment is 30 patients. In keeping with its exploratory design, the trial does not include a formal sample-size calculation: the sample size was chosen on the basis of comparable exploratory studies, with the aim of estimating outcome parameters with sufficient precision to inform a subsequent confirmatory trial. The present interim analysis was not foreseen in the study protocol. No formal interim stopping rules and no statistical criteria for study discontinuation were specified.

#### 2.1.1. Scope of the Present Report

Enrolment is ongoing and this analysis does not address the trial’s registered primary efficacy endpoint as a confirmatory result. The report is confined to the technical feasibility of the procedure and to procedural safety in the first 10 treated patients, with clinical measures presented descriptively. We are publishing this interim analysis for three reasons. First, to our knowledge this is the first use of non-adhesive liquid embolic agents for this indication, and the anatomical findings—above all the high frequency of anastomoses with the ophthalmic artery—bear directly on the safety of other groups beginning to perform MMA embolization for migraine. Second, the procedural observations from the first 10 patients—the relationship between branch tortuosity and catheter choice, and the need for superselective angiography before embolization—have informed the pre-procedural planning of subsequent cases within the technique specified in the protocol. Third, the analysis involves no between-group comparison, and no change to the protocol, the eligibility criteria, or the planned analyses was made on the basis of it. This report makes no claim of efficacy. The relationship between the trial endpoints and the outcomes reported here is set out in Section 2.6, and departures from the planned conduct of the trial are declared in Section 2.9. The registered primary efficacy endpoint will be reported when enrolment is complete, and all patients have reached the 48-week visit.

#### 2.1.2. Study Periods and Visits

Participation lasts no longer than 381 days per patient and comprises a 30-day screening period (Day −30 to Day −1), a one-day treatment period (Day 1), and a 48-week (±14 days) follow-up period. Nine visits are scheduled: screening (Visit 1, Day −30 to −3), hospitalization (Visit 2, Day −2 to −1), MMA embolization (Visit 3, Day 1), in-hospital assessment before discharge (Visit 4, Day 2–3), and follow-up at 4, 12, 24, 36, and 48 weeks (Visits 5–9). Unscheduled visits, including additional MR angiography, brain MRI or EEG, could be arranged by the investigator for further safety assessment.

#### 2.1.3. Headache Diary

Patients completed a daily headache diary recording the presence or absence of headache, attack duration, peak intensity on a numerical rating scale, associated symptoms, and acute medication use. The diary was issued at Visit 1 and completed for the 28 days preceding the procedure to establish baseline headache frequency, and continuously thereafter; a diary covering the interval to the next visit was issued at each visit and reviewed by the investigator on return.

#### 2.1.4. Diagnostic Framework

The study follows the recommendations of the International Headache Society for controlled trials of preventive treatment in chronic migraine: study periods are defined in weeks, one month is taken as four weeks, and the modified diagnostic threshold of at least 14 headache days and at least 8 migraine days per 28 days is applied in place of the standard “at least 15 headache days per month”.

#### 2.1.5. Ethics

The study was conducted in accordance with the Declaration of Helsinki and ICH GCP guidelines. Study protocol version 1.0 dated 1 October 2024 was approved by the Bioethical Committee of the International Higher School of Medicine, Bishkek, Kyrgyz Republic (Protocol No. 4, dated 17 October 2024) and the ethics committee of the L. G. Sokolov North-Western District Scientific and Clinical Center, Saint Petersburg, Russia (Protocol No. 1124, dated 27 November 2024). All patients provided written informed consent before the intervention and invasive study procedure. Note should be made for the first patient regarding the time delay between her granting consent for participation in the study and the end of his recording of the baseline data from his headache diary, which is provided in detail in Section 2.9.2. The trial is registered at ClinicalTrials.gov (NCT06848166, CHROME; https://clinicaltrials.gov/study/NCT06848166 (access on: 30 September 2026); first posted 26 February 2025). Registration took place after enrolment of the first participants (first patient treated 19 October 2024) and is therefore retrospective with respect to the ICMJE requirement for registration before enrolment of the first participant. The registered primary endpoint, secondary endpoints, planned sample size, and single-arm open-label design correspond to protocol version 1.0 as approved by both ethics committees and were not changed at registration or thereafter.

### 2.2. Eligibility Criteria and Patient Selection

Eligibility criteria were defined in protocol version 1.0 of 1 October 2024, under which all 10 patients reported here were enrolled. The complete list of criteria is provided in Table S1.

#### 2.2.1. Inclusion Criteria

Men and women aged 21–70 years at the time of consent were eligible if they had chronic migraine diagnosed at least 12 months before screening according to the International Classification of Headache Disorders, 3rd edition (2018) [23], and resistant migraine established at least six months before screening according to the European Headache Federation consensus criteria (2020) [6,24]—that is, failure of at least three classes of migraine preventive medication, together with failure of non-pharmacological preventive and therapeutic approaches. Prior treatment was required to include botulinum toxin type A administered according to the PREEMPT protocol without benefit in headache frequency or intensity. Eligibility further required at least 14 headache days during the 28-day screening period, of which at least 8 met the definition of migraine, documented prospectively in the daily headache diary; and a maximum headache intensity on migraine days of at least 5 points on an 11-point numerical rating scale during screening. Women of childbearing potential and men agreed to use adequate contraception throughout the study.

#### 2.2.2. Exclusion Criteria

Patients were excluded for any contraindication to MMA embolization identified at screening and for allergy to iodinated contrast media.

##### Neurological Exclusions

Neurological exclusions comprised cerebral vascular malformations, arteriovenous malformations, intracranial aneurysms, brain tumor, hydrocephalus, arachnoiditis, disorders of cerebrospinal fluid circulation, craniovertebral junction anomalies, platybasia, clivus deformity, severe traumatic brain injury, skull base fracture or its sequelae, previous neurosurgical procedures, stenosis or occlusion of the precerebral arteries, previous stroke or transient ischemic attack, epilepsy or seizures, and any neurological disease other than migraine.

Patient 9 had a subcutaneous occipital nerve stimulator for migraine implanted on the left side of her head in 2014 at another center. This device had not been activated for 2 years before screening for this study and remained switched off for the duration of the study. Imaging and angiography studies did not interfere with the device, and the extracranial implantation was without complication. Importantly, there were no persistent neurological deficits; as such, this patient did not meet the exclusion criterion for sequelae of neurosurgical procedures. Although somewhat of an outlier to the intended population of patients without prior invasive or neuromodulation treatments for migraine, a sensitivity analysis for the clinical endpoints is provided in Table S10 for Patient 9.

##### Headache-Specific Exclusions

Headache-specific exclusions were medication-overuse headache at screening or within the preceding 12 weeks; other chronic headache disorders of less than 12 months’ duration, including trigeminal autonomic cephalalgias, new daily persistent headache, painful cranial neuropathies, retinal migraine, aura without headache, hemiplegic migraine, migraine with brainstem aura, idiopathic intracranial hypertension without papilloedema, spontaneous intracranial hypotension and secondary headaches; inability of the patient to distinguish migraine from other headache types; complications of migraine (status migrainosus, persistent aura without infarction, migrainous infarction, migraine aura-triggered seizure); and migraine onset after the age of 50.

Medication-overuse headache is defined in ICHD-3 (code 8.2) by the frequency of acute medication use alone: headache on 15 or more days per month in a patient with a pre-existing headache disorder, with regular overuse for more than 3 months of simple analgesics on 15 or more days per month, or of triptans, ergotamines, opioids, combination analgesics, or multiple drug classes on 10 or more days per month. By this definition acute medication overuse is almost universal in chronic resistant migraine, and the European Headache Federation consensus regards its presence as compatible with resistant migraine [6]. Medication-overuse headache was nevertheless made an exclusion criterion because withdrawal of the overused medication can by itself substantially reduce headache frequency and would confound the assessment of the intervention. The criterion was therefore operationalized as headache attributable to medication overuse, defined by the response to a documented withdrawal attempt, rather than by the frequency of use alone. According to the routine practice of the participating centers, all candidates with acute medication use on 10 or more days per month underwent a structured withdrawal program 3–4 months before screening: abrupt discontinuation of the overused analgesics (gradual discontinuation in the one patient overusing tramadol), a bridging course of dexamethasone 8 mg/day intravenously for 5 days, antiemetics as required, and bilateral greater occipital nerve block with lidocaine in two patients; the overused class was not resumed during the bridging period. Headache frequency was reassessed from the diary approximately 8 weeks after withdrawal. A reduction in headache days of 50% or more was taken to indicate medication-overuse headache and led to exclusion; this applied to one candidate (27 to 9 headache days per 28 days). In the 10 enrolled patients, headache frequency was unchanged after withdrawal (22–28 days before versus 21–28 days after), acute medication use subsequently returned to the pre-withdrawal level and, by the ICHD-3 frequency thresholds, all 10 met the criteria for medication overuse during the 28-day screening period (Table S12). No further withdrawal was undertaken during the trial.

##### General Medical Exclusions

General medical exclusions comprised body mass index below 18.5 or above 39.9 kg/m^2^; metabolic syndrome or cachexia; psychiatric illness, including depression confirmed by a psychiatrist at screening on the basis of HADS and Hamilton assessment, judged likely to affect the patient’s assessment of their own condition or their safety in the study; substance or alcohol misuse; hyperthyroidism or diabetes mellitus; severe cardiovascular disease (NYHA class III–IV heart failure, clinically significant ventricular arrhythmia, significant valvular disease, uncontrolled hypertension above 180/110 mmHg, pulmonary embolism or deep vein thrombosis); varicose veins or previous lower-limb thrombosis; ischaemic heart disease or coronary atherosclerosis; stenosing atherosclerosis of the brachiocephalic arteries; atrial fibrillation, patent foramen ovale, atrial septal defect or previous cardiac surgery; coagulation disorders or antiphospholipid syndrome; anticoagulant or antiplatelet use within five days of the procedure; uncontrolled asthma or severe chronic obstructive pulmonary disease; estimated glomerular filtration rate ≤ 60 mL/min or a history of renal failure; hepatic impairment or elevated transaminases; generalized pain syndrome or fibromyalgia; positive serology for HIV, hepatitis B or C, or syphilis; malignancy within five years or current suspicion of malignancy; pregnancy or planned pregnancy within three months; and any other unstable or clinically significant condition judged by the investigator to preclude participation. Participation in another interventional trial within 30 days of screening, inability to read or write or to comply with study procedures, and employment at a participating site or a first-degree relationship with such an employee were also exclusions.

#### 2.2.3. Withdrawal Criteria

Patients were withdrawn on withdrawal of consent; on an adverse event preventing compliance with protocol procedures; if continued participation was judged by the investigator to be contrary to the patient’s interest; for major protocol violation or refusal or inability to comply with study procedures; or on loss to follow-up.

#### 2.2.4. Screening and Enrolment

Patients with chronic migraine were assessed for eligibility at the two participating centers between October 2024 and June 2025. Because of procedure scheduling, the interval between confirmation of eligibility under the European Headache Federation criteria and the procedure ranged from 1 to 7 months (median 2 months). Preventive and acute medication were unchanged between confirmation of eligibility and the procedure. Irrespective of this interval, every patient underwent the protocol-defined 28-day screening period and screening investigations within the window specified in the protocol (Day −30 to Day −3), with the exception of the timing of the baseline diary in the first patient described in Section 2.9.2, and eligibility—including the diary-based frequency and intensity criteria—was confirmed on the screening diary immediately preceding the procedure. The 10 patients reported here were treated between 19 October 2024 and 26 November 2025, and the data cut-off for this interim analysis was 5 September 2026.

A total of 318 patients with chronic migraine were assessed for eligibility across the two participating centers: 231 at the North-Western District Scientific and Clinical Center named after L. G. Sokolov, Federal Medical and Biological Agency, Saint Petersburg, Russia, and 87 at the Vedanta Medical Center, International School of Medicine, International University of Kyrgyzstan, Bishkek, Kyrgyzstan. Of these, 302 did not meet the European Headache Federation criteria for resistant migraine, and 16 (5.0%) did—11 of 231 (4.8%) in Saint Petersburg and 5 of 87 (5.7%) in Bishkek.

Eligibility assessment for participants at study sites was carried out by headache medicine neurologists of participating centers who were treating patients with migraine on an ongoing basis. These neurologists were not involved in the MMA-embolization interventions and were not part of the intervention’s technical assessment.

**Figure 1:**
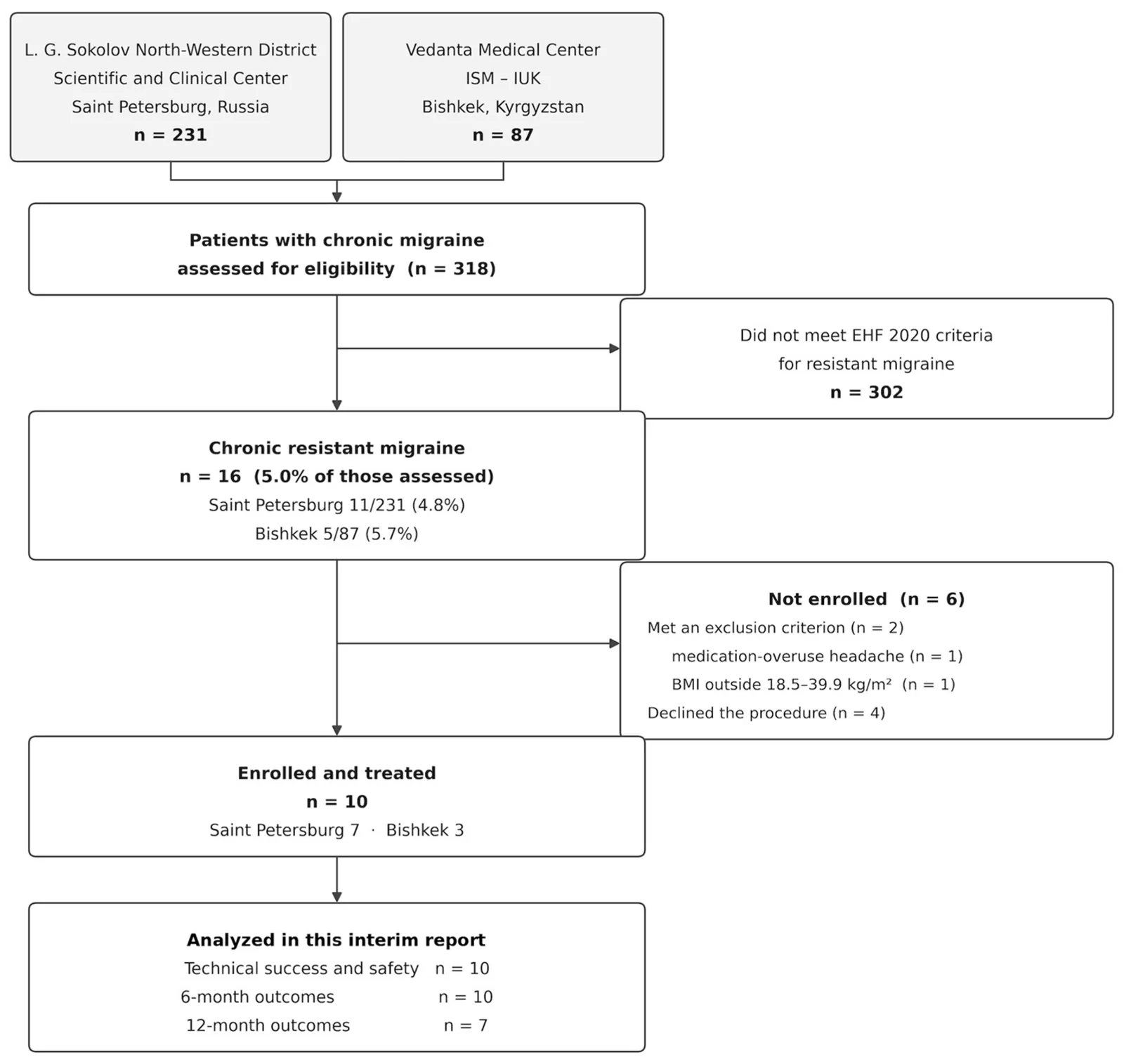
Flow of patients through screening, enrolment, and analysis, adapted from the CONSORT extension for randomized pilot and feasibility trials for a single-arm design. Patients with chronic migraine were assessed for eligibility at two participating centers between October 2024 and June 2025. Resistant migraine was defined according to the 2020 consensus criteria of the European Headache Federation. Of the 16 patients meeting these criteria, two were excluded at screening (one for medication-overuse headache, one for a body mass index outside 18.5–39.9 kg/m^2^) and four declined the procedure; the remaining 10 were treated and constitute the population of this interim analysis. Numbers analyzed differ between timepoints because enrolment and follow-up are ongoing: at the data cut-off of 5 September 2026, three patients had not yet reached the 12-month visit, and two had not yet completed the 48-week diary period. No patient was lost to follow-up. BMI, body mass index; EHF, European Headache Federation; ISM–IUK, International School of Medicine, International University of Kyrgyzstan.

Of these 16 patients, two were subsequently excluded at screening: one for medication-overuse headache—acute medication use on 26 of the 28 screening-diary days, with a history of regular overuse for more than 3 months, meeting ICHD-3 criteria (Section 2.2.2, Headache-Specific Exclusions)—who was referred for withdrawal treatment and was not re-screened within the enrolment period, and one for a body mass index outside the permitted range of 18.5–39.9 kg/m^2^. Four patients declined the procedure. The remaining 10 patients were enrolled and treated—7 in Saint Petersburg and 3 in Bishkek—and constitute the population of this interim analysis. The flow of patients through screening and enrolment is shown in Figure 1.

At baseline, no patient reported strictly unilateral headache: 7 patients reported bilateral headache and 3 reported side-shifting attacks. Bilateral embolization was therefore performed in accordance with the protocol, which specifies ipsilateral embolization only for unilateral pain localization. Mean duration of chronic migraine was 7.1 ± 2.3 years, and all patients had failed at least three classes of preventive medication as well as botulinum toxin type A.

### 2.3. MMA-Embolization Technique

The procedure aimed for bilateral embolization of both frontal and parietal MMA branches to maximize dural devascularization. All procedures were performed under general anaesthesia via a transfemoral approach. A 7-F femoral introducer (Avanti+, Cordis, Miami Lakes, FL, USA) was placed, and systemic heparinization (2500 IU per procedure) was given. Diagnostic angiography, performed in every patient before embolization as required by the protocol, included selective injections of both internal carotid arteries, both external carotid arteries and the vertebrobasilar system, followed by superselective injection of each target MMA branch; the internal carotid injection was specifically reviewed for an ophthalmic origin of the MMA and for MMA–ophthalmic anastomoses (Section 2.4). A triaxial system was used for catheterization: a 6-F coaxial guiding system (Chaperon, 6-F guide with 5-F inner catheter, MP2 shape; MicroVention, Aliso Viejo, CA, USA) or a 7-F Envoy guiding catheter (Cerenovus, Irvine, CA, USA) was positioned in the external carotid artery, and the microcatheter was advanced over a 0.014-inch microguidewire (Chikai, Asahi Intecc, Nagoya, Japan). The microcatheter was selected according to the diameter and tortuosity of the branch (Section 3.2): a 1.5-Fr detachable-tip microcatheter (Apollo, Medtronic Neurovascular, Irvine, CA, USA), a 1.7, 2.1, or 2.7-Fr microcatheter (Headway 17, 21 or 27; MicroVention), or a double-lumen DMSO-compatible balloon microcatheter (Scepter, MicroVention; 3 × 8, 3 × 10 or 3.5 × 10 mm). When the MMA or one of its branches arose from the ophthalmic artery, that branch was not catheterized for embolization, in accordance with the safety rules of the protocol (Section 2.4 and Section 3.2).

For the widest possible distribution and safe penetration of the non-adhesive liquid embolic agents (NLEAs) without embolization of dangerous anastomoses, a wedge technique was used or, when this was not possible, double-lumen dimethyl sulfoxide (DMSO)-compatible balloon microcatheters (Scepter, MicroVention, Aliso Viejo, CA, USA). Selective angiography with a 1-mL syringe confirmed the wedge position or balloon inflation and the presence or absence of reflux. Reflux on the initial microangiography was present in 7 of the 29 branches in which it could be assessed and absent in 22 (Table S2); where present, a proximal plug of SQUID 18 was formed first to control the reflux, after which SQUID 12 was injected for distal penetration. Otherwise, embolization was performed with a combination of NLEAs of different viscosity in the reverse order: SQUID 12 first, for distal penetration, followed by SQUID 18 to control the spread of the agent. Overall, SQUID 12 was used in 24 and SQUID 18 in 26 of the 40 target branches (Table S2). SQUID 18 was used at the end of each injection for three purposes:

- to inhibit excessive distal penetration during retrograde spread of NLEA along the anterior and posterior meningeal arteries (since they are directly related to the ophthalmic and vertebral arteries, respectively);
- to inhibit penetration in the case of direct connections (“microfistulas”) between the MMA branches and the dural venous sinuses, and when the NLEA enters the venous lacunae of Trolard;
- to inhibit excessive spread through the dural venous system (for example, in cases of risk of NLEA entering from the sphenoparietal sinus into other venous collectors) to create a proximal plug and filling the arterial network of the frontal and parietal representations of the MMA.

### 2.4. Anatomical Factors for MMA Embolization

**Figure 2:**
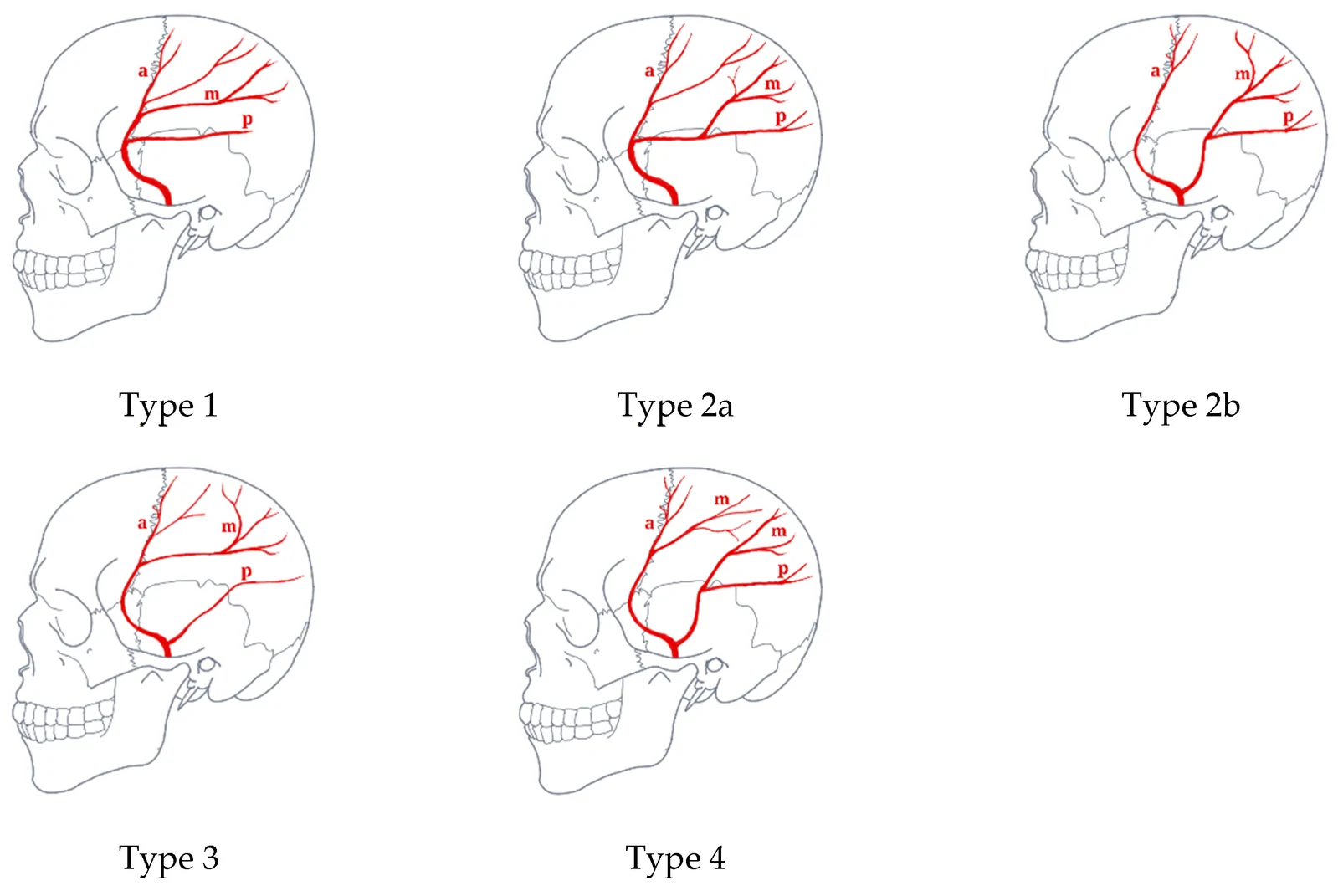
Schematic illustration of the different types of intracranial branches of the middle meningeal artery. a, m, and p denote the anterior, middle, and posterior branches. Type 1 is the pattern labeled as type 1 in the Adachi and Giuffrida–Ruggeri classifications. Type 2a is a pattern labeled as type 2 by Adachi and as type 2a by Giuffrida–Ruggeri. Type 2b, a model designated as type 2 by Adachi but as type 2b by Giuffrida–Ruggeri because of the low division. Type 3 is a pattern labeled as type 1 by Adachi, but as type 3 by Giuffrida–Ruggeri because of the low division of the artery into terminal branches. Type 4, a pattern labeled as type 3 by Adachi and type 4 by Giuffrida–Ruggeri [25,26,27,28,29].

**Figure 3:**
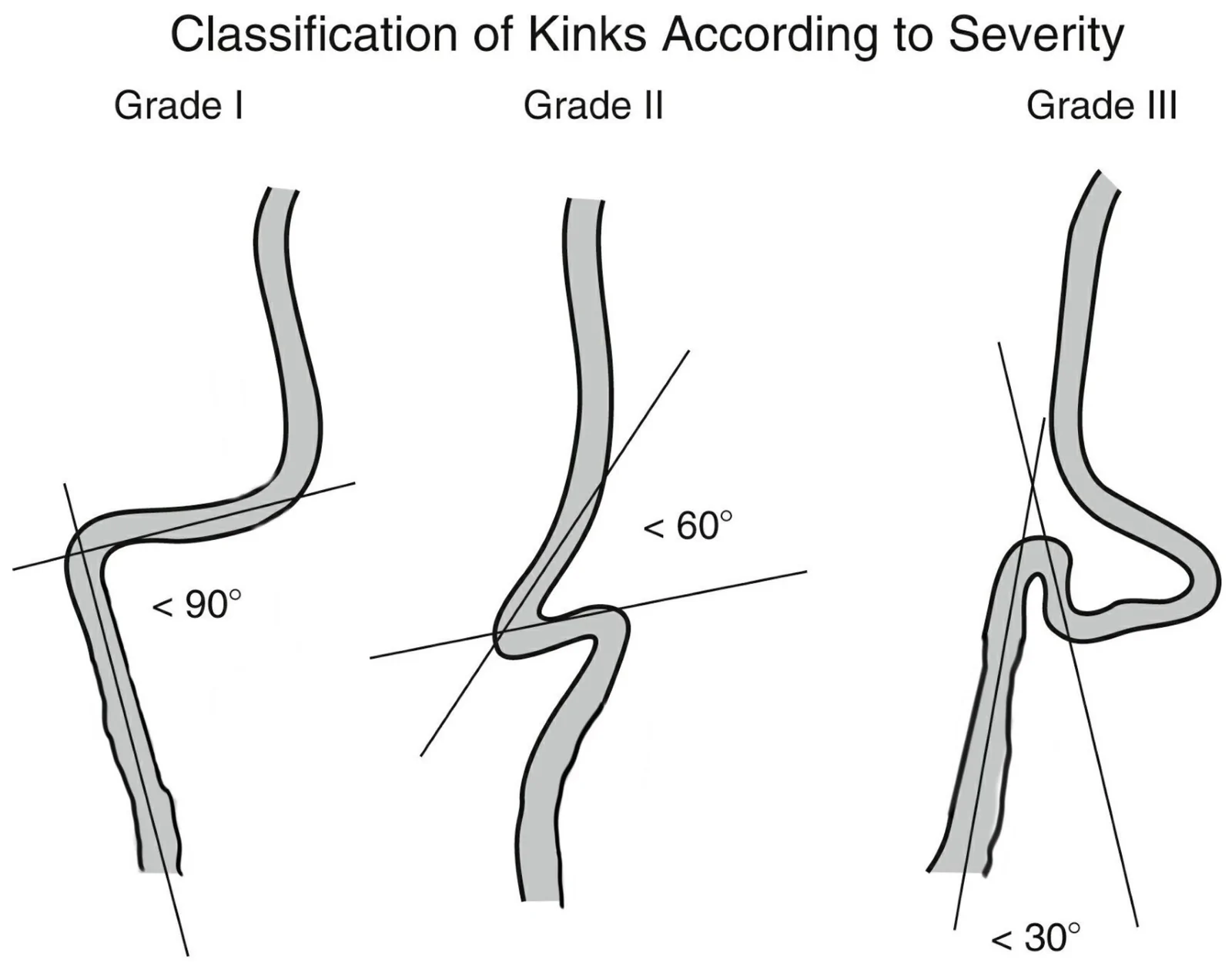
Schematic diagram of the three grades of tortuosity of the frontal and parietal MMA branches. Grades I–III correspond to increasing kinking and angulation of the branch; a branch without kinking or angulation was recorded as having normal tortuosity and is not shown.

The following anatomical factors, which influence both the planning and the technique of MMA embolization, were recorded in every patient according to the protocol:

- MMA structure variations according to Adachi (Figure 2);
- thorough detection of proximal (tympanic, cavernous, ophthalmic) and distal anastomoses (anterior meningeal, repeated ophthalmic collaterals, posterior meningeal artery); ectopia of the ophthalmic artery (originating from the MMA);
- the degree of kinking /angulation (Figure 3) of the MMA branches. The presence of these anatomical features was considered as factors determining the distal position of the catheter and the use of (wedge technique)/DMSO-compatible balloons.

### 2.5. Evaluation of the Degree of Embolization

**Figure 4:**
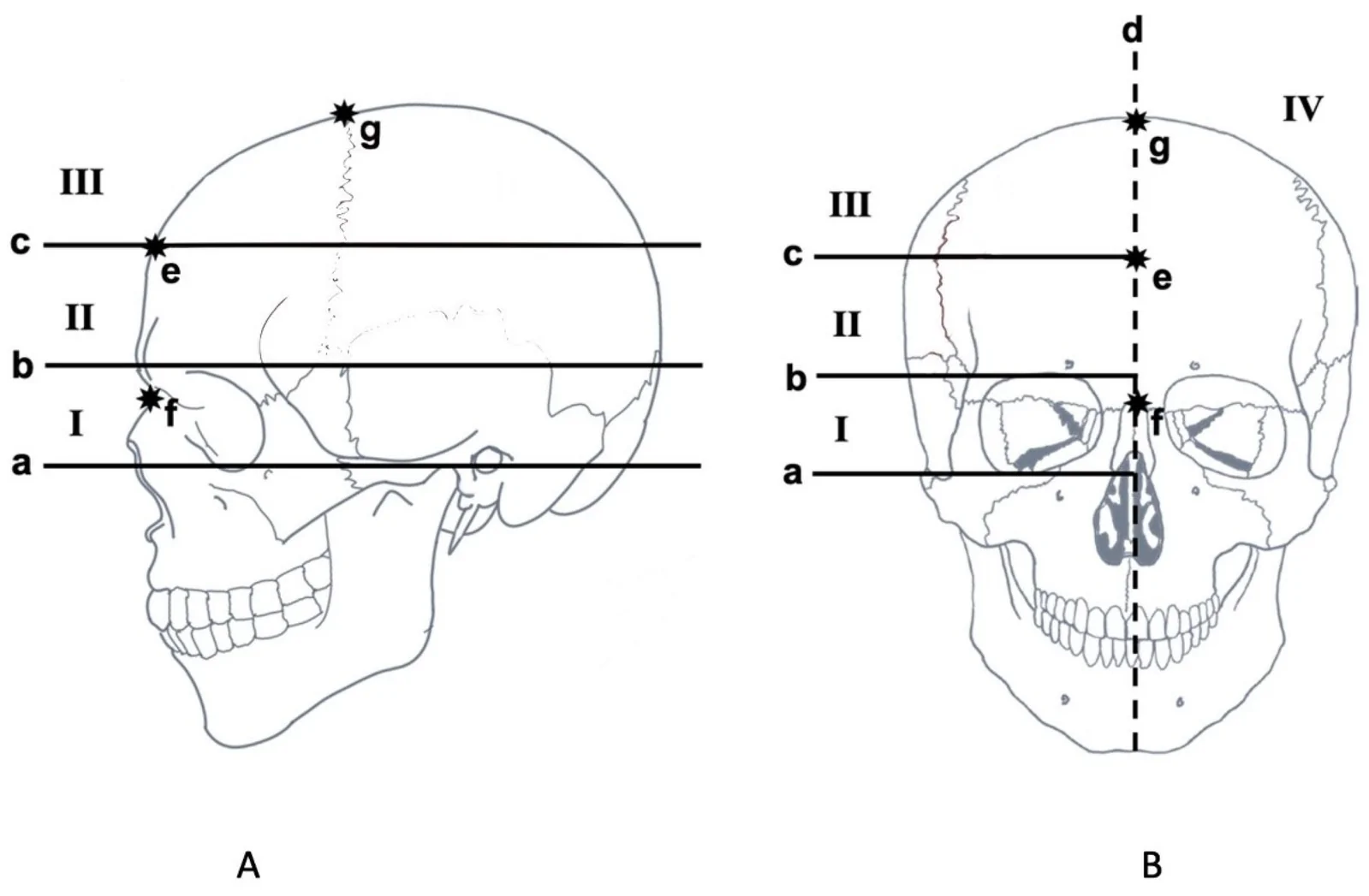
The schematic illustration of the approach used to assess the level of the DM vascularization. (**A**) lateral view of the skull; (**B**) frontal view of the skull. Stars mark the craniometric points e, f, and g. a—auriculo-orbitalis line (the projection of the Frankfort horizontal plane in frontal view); b—supra-orbitalis line (the plane’s projection in frontal view); c—the median point line, parallel auriculo-orbitalis and supra-orbitalis lines (the plane’s projection in frontal view); d—midline; e—the median point; f—Nasion; g—Bregma; I, II, III, IV—vascularization levels of the DM [30].

Dural embolization was assessed intraoperatively on craniograms immediately after embolic agent injection; the approach to assessing DM devascularization [30] is illustrated in Figure 4.

In the lateral projection on radiographs and DSA, three levels of vascularization of the ipsilateral DM were identified. For convenience in delineating the vascularization level, the Frankfort horizontal plane was used as a basis. The auriculo-orbitalis line passes through this plane in the lateral projection. The second line supra-orbitalis as in the scheme of R.U. Krönlein [31] runs parallel to the line auriculo-orbitalis. The third line was added by us to emphasize the most distal blood supply zone of the ipsilateral MMA; it also runs parallel to the first two lines and through the craniometric Median point. Median point, instrumentally determined on the frontal bone as the greatest elevation from a cord between Nasion and Bregma. Nasion point—intersection of the nasofrontal sutures in the median plane. Bregma point—where the sagittal and coronal sutures meet. The sagittal plane is the main boundary of the vascularization level of the ipsilateral MMA.

Thus, the vascularization levels of the ipsilateral MMA can be distinguished as follows:

- Level I or proximal, bounded by the auriculo-orbitalis and supra-orbitalis lines;
- Level II medial, bounded by the supra-orbitalis and a line through the craniometric Median point;
- Level III is parasagittal, limited by the line through the craniometric Median point and sagittal plane;
- Level IV—contralateral, reached through anastomoses with the contralateral meningeal arteries and the falcine arteries.

Level I devascularization: the main trunk of the MMA and the proximal segments of the second-order branches are occluded without penetration into the terminal branches; the bulk of the embolic agent lies within level I.

Level II devascularization: the second-order branches are occluded with partial penetration into the third-order branches; embolization is confined to levels I–II.

Level III devascularization: the embolic agent reaches the parasagittal vascularization zone of the MMA.

Level IV devascularization: the embolic agent spreads both into the ipsilateral target zone and to the contralateral side, with penetration into the falcine arteries and their anastomoses. Level IV devascularization was not a goal of the procedure: because the anterior falcine artery arises from the ophthalmic artery, spread of the embolic agent towards the midline was regarded as a safety limit, and injection was stopped as soon as the agent approached the falcine arteries or the contralateral dura on fluoroscopy.

Penetration of the embolic agent into the dural veins was recorded as an angiographic observation (present/absent). It was not a technical goal of the procedure. However, during the first procedures it became apparent that SQUID readily enters the dural veins, in some branches already with the first injection (5 branches; Table S2); thereafter, venous penetration was accepted as part of the embolization, with the constraint that the agent was not allowed to reach the dural venous sinuses—injection was stopped as soon as the agent approached a sinus on fluoroscopy, and a proximal plug of SQUID 18 was formed if necessary. Venous penetration is therefore reported both as a component of the degree of penetration (SPADES “+Vens” grades) and as a potential safety observation (Section 3.4 and Section 3.9).

The level of catheter tip placement (I–III; Table S8) refers to the position of the microcatheter before injection, whereas the level of devascularization (I–IV) describes the extent of spread of the embolic agent after injection; the two are related but not identical, since the agent may penetrate beyond the catheter tip or, in the case of reflux, remain proximal to it.

**Figure 5:**
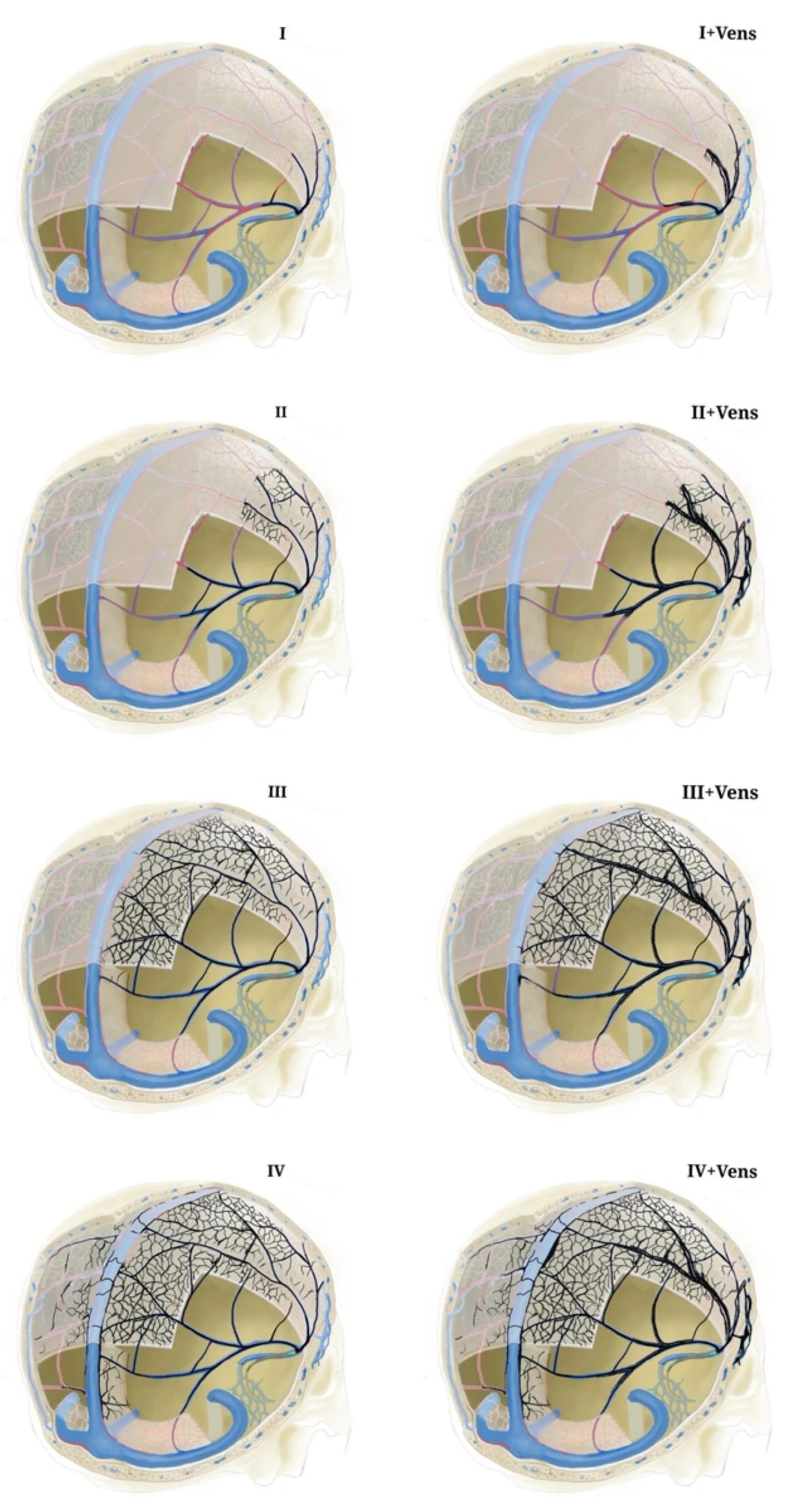
The Saint Petersburg Arterial Dural Embolization Scale (SPADES). The panels illustrate the progressive grades of embolic agent penetration through the dural arterial network ((**left column**), I–IV) and corresponding venous phase visualization ((**right column**), I+Vens–IV+Vens). The embolic agent cast is shown in black, the meningeal arteries in red, and the dural venous sinuses and veins in blue. Adapted from Shapiro M, et al., 2022, with permission from BMJ Publishing Group [32]. Original illustration by Jonathan Dimes.

From this approach, a newly proposed nine-grade scoring system—the Saint Petersburg Arterial Dural Embolization Scale (SPADES)—was formulated (Figure 5). It comprises grades I–IV, each with or without venous extension (eight grades); target branches that were not embolized were additionally assigned grade 0, so that nine categories were used in the analysis. Applicable to individual branches or the cumulative effect, it builds on the dural angioarchitectural concepts described by Shapiro et al. [32].

Grades were assigned by the operator from the intraoperative craniograms and the final angiographic run and were independently reviewed by a second reader; grade III or higher in a branch was taken, post hoc, as the definition of technical success for this interim analysis (Section 2.6.2). SPADES is an operator-assessed anatomical description of embolic penetration developed for this trial; it is not a validated outcome instrument, and it is used only to characterize technical results.

### 2.6. Endpoints

#### 2.6.1. Trial Endpoints

The registered primary-efficacy endpoint of the trial is the change from baseline (screening period) in the number of migraine days over the preceding four weeks, assessed 12 weeks after embolization. Secondary efficacy endpoints are monthly migraine days and monthly headache days at 4, 8, 12, 24, 36, and 48 weeks; the proportions of patients achieving reductions of ≥30% and ≥50% in monthly migraine days and ≥50% in monthly headache days at each timepoint; the number of days with moderate or severe headache, defined as headache of moderate or severe intensity lasting at least four hours; maximum headache intensity during attacks on an 11-point numerical rating scale; the number of days with associated symptoms (nausea, vomiting, photophobia, phonophobia); and total scores and changes from baseline on the Headache Impact Test-6 (HIT-6) and the Migraine Disability Assessment (MIDAS). Additional endpoints are depression severity assessed with the Hospital Anxiety and Depression Scale (HADS) and the Hamilton Depression Rating Scale. Safety endpoints are defined in Section 2.7.

The data from the headache diaries of the participants, as well as their scores on the MIDAS, HIT-6, and HADS questionnaires were collected and verified by the same set of headache experts from the participating centers. These experts were different from the operators and thus had not performed the embolization of the patients included in the trial. Because the trial was a single-arm, open-label study, these assessors were aware that all patients included in the trial had been treated by embolization and thus no independent third-party or blinded assessment of outcome was performed. Outcome on angiography (SPADES grading) was assessed by the operator and a second reader as described in Section 2.5.

#### 2.6.2. Outcomes of the Present Interim Analysis

Because enrolment is incomplete and follow-up is ongoing, this report does not evaluate the trial’s primary efficacy endpoint as a confirmatory result. The main outcome of this interim analysis—defined post hoc for this report and not a registered trial endpoint—is technical success, defined as achievement of SPADES grade III or higher (III, III+veins, IV, IV+veins) in a target MMA branch. Technical success is reported at two levels: at the branch level, with all 40 target branches in the denominator on an intention-to-treat basis (branches that could not be embolized are counted as failures), and at the patient level, as grade III or higher in all four target branches. Secondary outcomes of this analysis are the frequency and location of MMA anastomoses, the distribution of embolic penetration by branch, the relationship of branch diameter and tortuosity to the device used, and procedural safety.

SPADES grades were assigned by the operator at the time of the procedure and entered into the electronic case report form. All 40 branches were subsequently re-scored by a second interventional neuroradiologist blinded to clinical outcome; the raw grades of both readers are given in Table S13. The second reader disagreed with the operator in 4 of 40 branches (three on venous extension, one on arterial level); in all four the consensus grade used in the analyzis (Section 3.2) corresponded to the operator’s grade. Quadratic-weighted Cohen’s κ between the two readers was 0.98 (95% CI 0.89–1.00) for the full nine-category scale and 0.99 (95% CI 0.92–1.00) for the arterial level alone; unweighted κ for venous extension was 0.83 (95% CI 0.55–1.00). Confidence intervals were obtained by bootstrap resampling of patients (10 000 replicates) to account for the clustering of four branches within each patient. Using the second reader’s grades instead of the consensus, technical success would have been 30 of 40 branches and 5 of 10 patients (versus 29 of 40 and 4 of 10), so the conclusions do not depend on the adjudication.

#### 2.6.3. Clinical Measures Reported Descriptively

Monthly migraine days, monthly headache days, MIDAS, HIT-6, and HADS are reported for the patients who reached each timepoint, in order to describe the clinical course of this cohort and to avoid selective reporting of the trial’s outcome measures. These data are exploratory. They derive from an unblinded, uncontrolled cohort of 10 patients with incomplete follow-up, they are not adjusted for multiplicity, and they cannot be distinguished from a placebo response; they are presented as hypothesis-generating and support no conclusion about efficacy.

#### 2.6.4. Concomitant Medication

Preventive medication being taken at enrolment (patients 1, 3, 6, and 10; Table S11) was continued unchanged throughout follow-up. No new preventive medication was started in any patient during the 48-week follow-up, with the exception of trazodone prescribed for insomnia in patient 1. Acute medication was taken as needed and recorded in the daily diary; days with acute medication per 28-day period are reported in Table S12.

### 2.7. Safety Assessment

Safety was assessed via clinically significant deviations in vital signs; laboratory data (blood tests, coagulation, urinalysis); physical, neurological, and ophthalmological examinations; and brain CT and MRI (including DWI) within 48 h postoperatively to detect ischemic complications. MRI and MR angiography were repeated if adverse events were suspected.

Ophthalmological examination (visual acuity, ophthalmoscopy, and visual fields) was performed before the procedure and before discharge. Cranial nerve function—including the facial nerve, which is relevant because of the petrous branch of the MMA—and the condition of the scalp and skin and of the access site were assessed before discharge and at every follow-up visit. All adverse events were recorded with their seriousness, relationship to the procedure and duration, in accordance with the CONSORT extension for harms. Follow-up cerebral angiography was scheduled by the protocol at 12–15 months to assess the durability of occlusion and the state of the dural veins reached by the embolic agent, since this information cannot be reliably obtained non-invasively after embolization with a radiopaque agent; it was performed only after separate written informed consent and included a venous phase (Section 3.4).

### 2.8. Statistical Analysis

Statistical analysis was performed with StatTech v.4.16.1 (StatTech LLC, Moscow, Russia). Data are presented as mean ± SD, median (IQR) or n (%, 95% CI). Because of the small sample, MIDAS, HIT-6, and HADS are summarized as mean ± SD with 95% CI for descriptive purposes and compared across the six timepoints with the Friedman test; categorical variables are compared with the Fisher exact test. Longitudinal analyses include only patients who completed the corresponding visit, and the number analyzed is stated for every timepoint; the longitudinal analyses of MIDAS, HIT-6, and HADS comprise the 7 patients with 12-month data, so their baseline values differ from those of the whole cohort in Table 1 (n = 10). No adjustment for multiplicity was applied; all *p*-values are descriptive and exploratory, and no confirmatory inference is intended. Missing data were not imputed. Binomial 95% CIs were calculated by the exact Clopper–Pearson method. Associations at the level of branches and arteries (Section 3.2 and Section 3.3) concern 30–40 branches clustered within 10 patients; they are therefore presented descriptively, and where a *p*-value is given it derives from a permutation test that preserves the clustering of branches within patients. Spearman correlations are reported with 95% CIs obtained by bootstrap resampling of patients (10,000 replicates). Quartiles were calculated by linear interpolation between order statistics (Hyndman–Fan type 7), which yields fractional quartile boundaries for integer diary counts.

### 2.9. Changes to Planned Outcomes

The relationship between the trial’s planned outcomes and those reported here is set out below in accordance with CONSORT item 6b.

#### 2.9.1. Reporting of Outcomes

The trial’s registered primary efficacy endpoint—change from baseline in monthly migraine days at 12 weeks—is not evaluated as a confirmatory result in this interim report, because enrolment is incomplete. It is reported descriptively in Section 3.5 and will be formally analyzed in the final report. The main outcome of the present analysis, technical success by SPADES, is an additional measure specific to this interim report; it was defined post hoc for this report (Section 2.6.2) and was not among the trial’s registered endpoints. No registered outcome was removed or changed, and the registry entry (NCT06848166) has not been modified in respect of outcomes since first posting.

#### 2.9.2. Individual Deviations

One protocol deviation occurred, affecting the first patient enrolled (patient 1, Bishkek). The protocol specifies that the 28-day baseline headache diary is issued at the screening visit (Day −30) after written informed consent. This patient had been under the routine care of the participating headache neurologist before the trial opened and had been keeping a prospective daily headache diary in the same format as part of that care. The 28-day diary period used for baseline assessment began on 16 September 2024 and ended on 13 October 2024; it was thus completed entirely before written informed consent was signed on 18 October 2024 and before the local ethics committee approved the protocol on 17 October 2024. The diary entries were not modified retrospectively. The screening investigations required by the protocol were also performed as part of routine clinical care within the protocol’s validity. They were available at the time of consent. Written informed consent, obtained before hospitalization and before any invasive procedure, explicitly covered the use of these pre-existing diary data for baseline assessment. No patient was hospitalized or treated before ethics approval had been granted at the relevant center. Because the diary was prospective, unchanged in format and not modified retrospectively, the baseline data of this patient were retained in the analysis and no sensitivity analysis excluding them was considered necessary.

Three further points were reviewed. First, patient 9 had an occipital nerve stimulator implanted in 2014, inactive since 2023; this was judged not to constitute a deviation, because the exclusion criterion refers to sequelae of neurosurgical procedures rather than to the procedures themselves (Section 2.2.2, Neurological Exclusions), and a sensitivity analysis excluding this patient is given in Table S10. Second, the follow-up angiography scheduled by the protocol at 12–15 months had not yet fallen due in patients 8, 9, and 10 at the data cut-off; this is a consequence of the interim timing, not a deviation. Third, in three of the seven patients who underwent follow-up angiography it was performed outside the protocol window—early, at 7 months, at the patient’s request because of persisting attacks, and late, at 16 and 18 months, because of work commitments (Section 3.4); these timing deviations are recorded as such. No other individual protocol deviations were identified.

## 3. Results

### 3.1. Characteristics of Patients

Of 318 patients with chronic migraine assessed for eligibility at the two participating centers, 16 (5.0%; 95% CI 2.9–8.0) met the European Headache Federation criteria for resistant migraine, and 10 were enrolled and treated. Of the six not enrolled, one was excluded for medication-overuse headache, defined by a marked response to the pre-screening withdrawal program (Section 2.2.2, Headache-Specific Exclusions), one for a body mass index outside the permitted range, and four declined the procedure after full information. The flow of patients through screening and enrolment is shown in Figure 1. The proportion meeting the resistance criteria was closely similar at the two centers despite differences in referral pattern and healthcare setting—eleven of two hundred and thirty-one (4.8%; 95% CI 2.4–8.4) in Saint Petersburg and five of eighty-seven (5.7%; 95% CI 1.9–12.9) in Bishkek. The ten treated patients (one man and nine women) were aged 24–67 years (mean 41.4 ± 13.7). Baseline characteristics are given in Table 1.

In addition to being on oral prophylactics that failed in three or more different classes, four patients had received an anti-CGRP monoclonal antibody (erenumab in three patients, fremanezumab in two patients) with transient relief but no lasting benefit. Four other patients were on an oral prophylactic at study entry which failed to give adequate control and thus was continued as was. All 10 patients met criteria for acute medication overuse (Section 2.2.2, Headache-Specific Exclusions, Table S12) as they had all failed a documented attempt to withdraw medications 3–4 months prior to study entry and met ICHD-3 frequency criteria for such overuse.

### 3.2. Technical Results of MMA Embolization and Anatomical Factors

All ten patients underwent MMA embolization—bilateral in nine, unilateral (right-sided) in one. Bilateral frontal and parietal branches were identified in all patients (40 branches total), of which 30 were directly catheterized.

**Figure 6:**
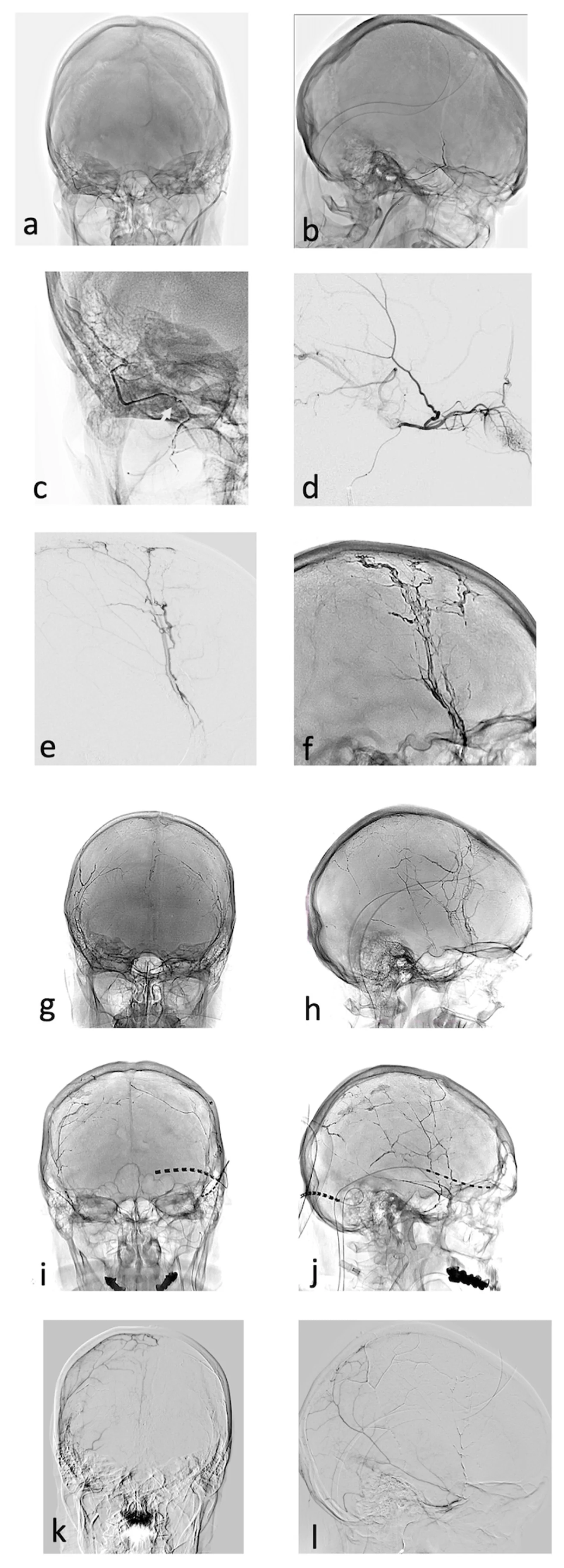
Representative cases demonstrating embolic agent distribution and anatomical characteristics in patients with CRM. Patient 5 (**a**–**d**): Anteroposterior (**a**) and lateral (**b**) projections; magnified anteroposterior projection (**c**); and DSA of the left ophthalmic artery (**d**) performed via left internal carotid- artery access. This case illustrates unilateral embolization, as the left side was excluded due to anatomical constraints. The distribution of the embolic agent in the right MMA was limited by the tortuosity of the MMA trunk. Patient 7 (**e**,**f**): Lateral DSA of the right MMA (**e**) and post-embolization SQUID cast (**f**). These images demonstrate dural arteriovenous connections identified during microangiography (**e**), and the subsequent distribution of the embolic agent through both arterial and venous networks via these connections (**f**). Patient 4 (**g**,**h**): Anteroposterior (**g**) and lateral (**h**) projections demonstrating the NLEA cast extending into both dural venous and arterial systems bilaterally. Patient 9 (**i**,**j**): Anteroposterior (**i**) and lateral (**j**) projections showing the non-adhesive embolic cast in a patient with a previously implanted left-sided nerve stimulator for migraine treatment. Patient 3 (**k**,**l**): Anteroposterior (**k**) and lateral (**l**) projections from a 12-month follow-up DSA. The patient reported incomplete pain resolution; selective microangiography of the right parietal MMA branch provoked a migraine attack on the angiography table.

Unfavorable anatomy prevented the embolization of four of the 40 branches: in one case, in which embolization was performed only on the right side, the left frontal and parietal branches of the MMA (two branches) arose directly from the left ophthalmic artery (Figure 6), and therefore, their catheterization was limited by the safety protocol of our study.

**Figure 7:**
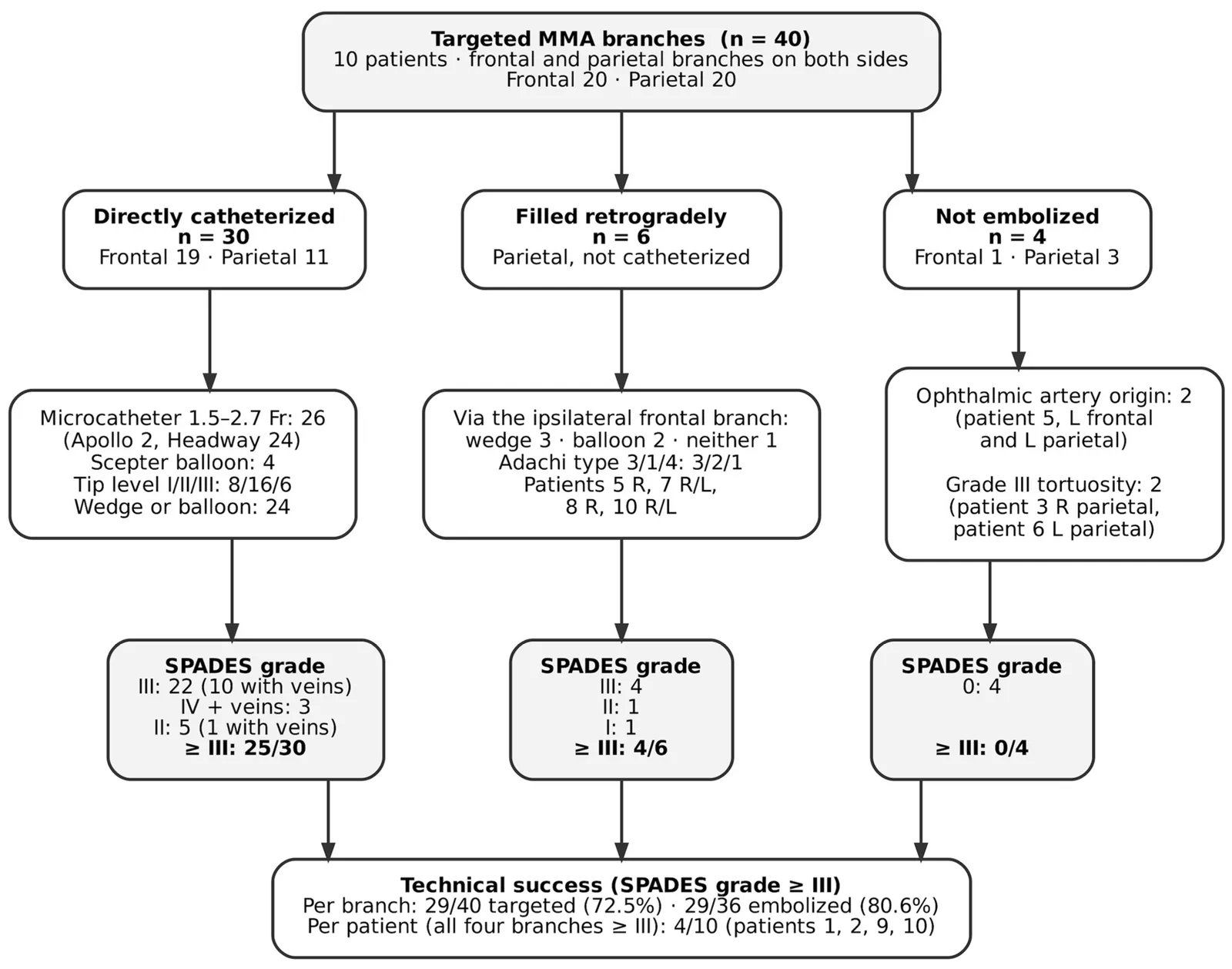
Fate of the 40 target MMA branches. Branches were catheterized directly, filled retrogradely from the ipsilateral frontal branch, or not embolized; the distribution of SPADES grades is given for each group (“with veins” denotes venous extension of the embolic agent). Technical success was defined as SPADES grade ≥ III; patient-level technical success required grade ≥ III in all four target branches. L, left; R, right; SPADES, Saint Petersburg Arterial Dural Embolization Scale.

In two further patients the parietal branch (two branches; patients 3 and 6) was not catheterized because grade III tortuosity of a small-caliber vessel (0.8 and 0.7 mm, respectively) did not allow the microcatheter to be advanced safely. Another six parietal branches, although not directly catheterized, were devascularized by retrograde filling of the parietal territory during embolization of the frontal branch. Retrograde filling reflected baseline anatomy: of these six branches, three arose from Adachi type-3 arteries, two from type 1 and one from type 4—configurations in which the parietal branch is naturally less prominent (Table S2). An extensive network of second- and third-order interbranch anastomoses, combined with the penetrating properties of the NLEA and embolization of the ipsilateral frontal branch in a wedge position (three branches) or through a DMSO-compatible balloon microcatheter (two branches), achieved devascularization of the parietal territory without direct catheterization (grade III in four branches and grade II in one). In the remaining branch (patient 5) the frontal branch was embolized without a wedge position or balloon, and retrograde filling reached only grade I. The fate of all 40 target branches is shown in Figure 7: 30 were catheterized directly (nineteen frontal, eleven parietal); six parietal branches, including the right parietal branch of patient 5, were filled retrogradely; and four were not embolized (the two left-sided branches of patient 5, arising from the ophthalmic artery, and the parietal branches of patients 3 and 6). SPADES grades were as follows: catheterized branches—grade III in 22 (ten with venous extension), IV with venous extension in three and II in five (one with venous extension); retrogradely filled branches—III in four, II in one and I in one; non-embolized branches—zero in four.

The distribution of penetration levels of the NLEA according to SPADES for 40 branches is presented in Table 2.

Grade III was the most frequently achieved level (26 branches; 65.0%; 95% CI 48.3–79.4), 10 of them with venous extension. Grade IV with venous extension was reached in three branches (7.5%; 95% CI 1.6–20.4). Grade II was recorded in six branches, one with venous extension. In one parietal branch (patient 5), which was not catheterized and is one of the six retrogradely filled parietal branches, retrograde filling from the ipsilateral frontal branch (grade II) reached only proximal grade I, and contralateral embolization was precluded by anatomy (scored grade 0). Two further non-catheterized parietal branches were also scored grade 0 (patients 3 and 6).

Thus, on an intention-to-treat basis, SPADES grade III or IV was achieved in 29 of 40 target branches (72.5%; 95% CI 56.1–85.4), corresponding to 29 of 36 embolized branches (80.6%; 95% CI 64.0–91.8). At the patient level, grade III or higher in all four target branches (patient-level technical success) was achieved in four of ten patients (40%; 95% CI 12.2–73.8; patients 1, 2, 9, and 10). In eight patients the embolic agent extended beyond the arteries into the venous drainage of the dura mater, bilaterally in five of them (14 branches; Table S14). The venous structures reached were the meningeal companion veins of the embolized branch in twelve of the fourteen branches, the lacunae of Trolard in five, the wall of the sphenoparietal sinus in four, and of the superior sagittal sinus in one (five branches in four patients), and diploic venous channels in three. The embolic agent did not enter the lumen of any dural sinus or any cortical vein. Venous spread was the reason for stopping the injection in six of the fourteen branches. On the venous phase of follow-up angiography, available for eight of these branches in five patients, all venous structures reached by the agent and the adjacent sinuses were patent.

MMA branch diameters ranged from 0.50 to 2.10 mm (median 0.95; IQR 0.80–1.40). Device selection was dictated by both diameter and tortuosity. A 1.5-Fr detachable-tip microcatheter (Apollo, Medtronic Neurovascular, Irvine, CA, USA) was used in two of the thirty catheterized branches (6.7%; 95% CI 0.8–22.1), a 1.7-Fr microcatheter (Headway 17, MicroVention, Aliso Viejo, CA, USA) in ten (33.3%; 95% CI 17.3–52.8), a 2.1-Fr microcatheter (Headway 21, MicroVention) in nine (30.0%; 95% CI 14.7–49.4), a 2.7-Fr microcatheter (Headway 27, MicroVention) in five (16.7%; 95% CI 5.6–34.7), and a dual-lumen balloon microcatheter (Scepter, MicroVention; 3 × 8 to 3.5 × 10 mm) in four (13.3%; 95% CI 3.8–30.7). The Apollo microcatheter was used in patient four (left parietal branch, grade III) and patient 8 (left frontal branch, grade III with venous extension); in both branches the detachable tip was detached and left within the embolic cast, so that these two patients received the liquid embolic agent together with a retained solid component. Patient 4 achieved a reduction in monthly migraine days of at least 50% by week 12, whereas patient 8 did not (0% at week 12; 10.0% at week 48); neither experienced a serious adverse event.

We assessed the relationship between branch tortuosity and microcatheter outer diameter, including balloon use (Table S3). Balloon microcatheters were used only in branches with grade III tortuosity. Pronounced tortuosity was associated with the use of either a balloon microcatheter or a smaller 1.5–1.7-Fr microcatheter, which provided the trackability needed to navigate beyond the tortuous segments: a balloon or a 1.5–1.7-Fr microcatheter was used in six of eleven branches with grade II–III tortuosity versus ten of nineteen with normal or grade I tortuosity (Table S3; permutation *p* = 0.084 with clustering of branches within patients preserved).

The level of tip placement was related to branch tortuosity rather than to the Adachi configuration. No branch with grade II or III tortuosity was catheterized beyond Level II, and six of these eleven branches were catheterized only to Level I, whereas all six branches reaching Level III had normal or grade I tortuosity (Spearman ρ = −0.50, 95% CI −0.82 to −0.07 by bootstrap resampling of patients). Branch diameter was not associated with the level reached (ρ = 0.18). Balloon microcatheters were used in four frontal branches, all with grade III tortuosity, and were positioned at Level I in three of them (Table S8).

The Adachi configuration was instead related to whether the parietal branch could be catheterized at all: none of the four arteries of types 3 or 4—the configurations with a low division and a small parietal branch—allowed catheterization of the parietal branch, compared with 11 of 16 (68.8%) of the remaining arteries. With four arteries in the first group, this contrast is reported descriptively. Consistent with this, of the six parietal branches devascularized by retrograde filling without direct catheterization, three arose from type 3 arteries, two from type 1 and one from type 4.

### 3.3. Frequency of MMA Anastomoses

MMA anastomoses requiring a special embolization technique were assessed preoperatively by selective cerebral angiography in all 10 patients (20 MMAs). An anastomosis with the ophthalmic artery was defined as any angiographically visible connection between the MMA and the ophthalmic territory: the recurrent meningeal (sphenoidal) artery through the superior orbital fissure, the meningolacrimal artery through the foramen of Hyrtl, and other connections considered.

Anastomoses of any location were identified in 17 of the 20 arteries studied (85.0%; 95% CI 62.1–96.8): proximal anastomoses in 10 (50.0%; 95% CI 27.2–72.8) and distal anastomoses in 14 (70.0%; 95% CI 45.7–88.1) (Table S4). The frequency of connections between the MMA, both proximal and distal, and the ophthalmic artery was noteworthy: they were found in 14 of the 19 MMAs available for evaluation (73.7%; 95% CI 48.8–90.9). One MMA could not be assessed because in patient 5 the left MMA branches themselves arose from the ophthalmic artery, so that an anastomosis between the two could not be identified separately; these branches were not embolized (Section 3.2). Distal and ophthalmic anastomoses (70.0% and 73.7% of MMAs) therefore need to be considered in planning, as they increase the risk of complications and may limit the intended distribution of the NLEA.

**Figure 8:**
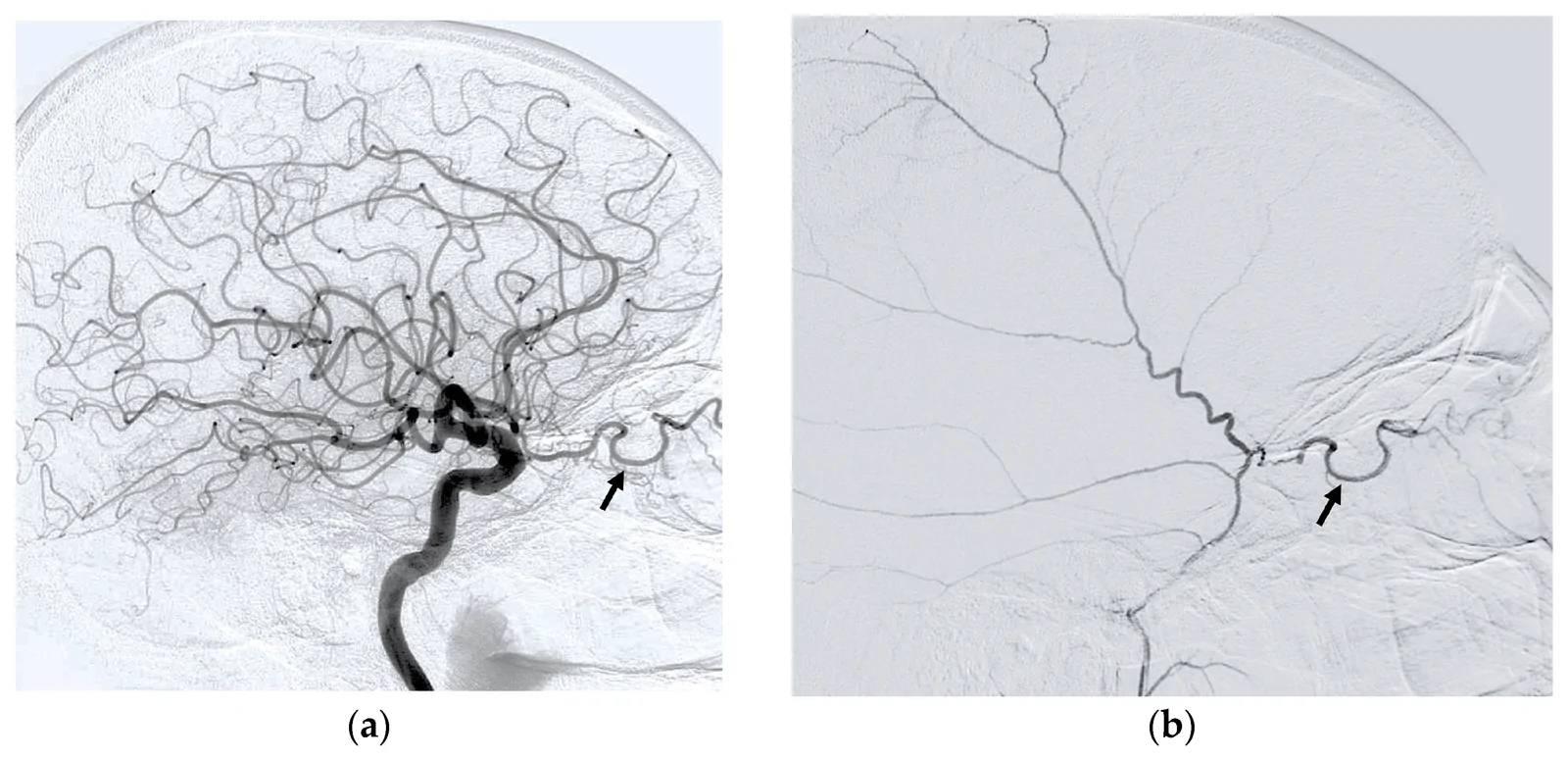
Digital subtraction angiography, lateral projection, arterial phase (patient 6, right side). (**a**) Right internal carotid artery injection. (**b**) Superselective injection of the right MMA. The black arrow indicates the right ophthalmic artery opacified through a proximal dangerous anastomosis with the right MMA via the recurrent meningeal (sphenoidal) artery.

Anastomoses were also taken into account when choosing the catheter tip position. Level II was the most frequent position overall (16 of the 30 catheterized branches; 53.3%), followed by Level I (8; 26.7%) and Level III (6; 20.0%); Level III was used only after the risk of the embolic agent reaching distal anastomoses had been excluded. The distribution differed between the two branches: Level II predominated in the frontal branches (13 of 19; 68.4%), whereas in the parietal branches Level III (5 of 11; 45.5%) and Level I (3 of 11; 27.3%) were the most frequent positions (Table S8). Balloon microcatheters were positioned at Level I in three frontal branches and at Level II in one. This difference reflects the larger number of anastomoses arising from the frontal branch. A representative example is shown in Figure 8: in the presence of grade II tortuosity, a proximal anastomosis with the ophthalmic artery required more distal catheterization and continuous monitoring of the anastomotic zone during injection to prevent reflux of the embolic agent into the ophthalmic artery.

### 3.4. Angiographic Follow-Up

Follow-up digital subtraction angiography, scheduled by the protocol at 12–15 months to assess the durability of occlusion, was performed in seven of the ten patients after separate written informed consent had been obtained. Four were performed within the protocol window; one was performed early, at 7 months, at the request of a patient with persisting migraine attacks, and two were delayed beyond the window, to 16 and 18 months, because of the patients’ work commitments. The remaining three patients (8, 9, and 10, treated in October–November 2025) had not reached the 12-month time point at the data cut-off of 5 September 2026. No procedure-related complication occurred. No recanalization of the embolized arteries was seen in any patient, including the patient examined at 7 months because of persisting attacks (Section 3.5). All follow-up angiograms included a venous phase: the dural venous sinuses and the venous structures reached by the embolic agent were patent in all seven patients (Table S14), and there was no angiographic evidence of dural sinus or cortical venous thrombosis. In two patients, both non-responders, selective injection of the parietal branch that had not been embolized at the index procedure provoked a migraine attack immediately after contrast injection: in patient 3, injection of the right parietal branch was followed by visual aura and right-sided headache lasting 23 h; in patient 6, injection of the left parietal branch was followed by visual aura and left-sided headache lasting 8 h. Both attacks were ipsilateral to the injected branch, met ICHD-3 criteria for migraine with aura, resolved without specific treatment, and are reported among the adverse events in Section 3.9.

### 3.5. Monthly Migraine Days and Monthly Headache Days

Diary-derived variables were computed over consecutive 28-day periods anchored to the day of the procedure: days 1–28 (4 weeks), 57–84 (12 weeks), 141–168 (24 weeks), 225–252 (36 weeks), and 309–336 (48 weeks). Baseline was the 28-day screening period. A migraine day was defined according to ICHD-3; monthly headache days comprise migraine days together with days of non-migraine headache. All patients who had reached the corresponding period at the data cut-off contributed to that timepoint, and the number analyzed is stated throughout; no data were imputed (Table S9).

At baseline the median number of monthly migraine days was 20.5 (IQR 18–22.5; range 18–24) and the median number of monthly headache days was 25.5 (IQR 23.2–27.0). Monthly migraine days were 16.5 (IQR 15.2–18; n = 10) at 4 weeks, eight (IQR 0.2–17; n = 10) at 12 weeks, seven (IQR 0.8–17.5; n = 10) at 24 weeks, eight (IQR 0.2–17.2; n = 10) at 36 weeks and 11.5 (IQR 0–17.2; n = 8) at 48 weeks. The corresponding median changes from baseline were −3, −13, −14, −13, and −11 days. Summary values are given in Table 3.

At the registered primary-endpoint timepoint of 12 weeks the median change from baseline in monthly migraine days was −13 days (IQR −19.5 to −1.8; n = 10). A reduction of at least 50% in monthly migraine days was achieved by six of ten patients (60%; 95% CI 26–88) at 12 weeks and by four of eight (50%; 95% CI 16–84) at 48 weeks; the same patients met the 30% threshold at both timepoints. A reduction of at least 50% in monthly headache days was achieved by six of ten patients at 12 weeks and four of eight at 48 weeks.

**Figure 9:**
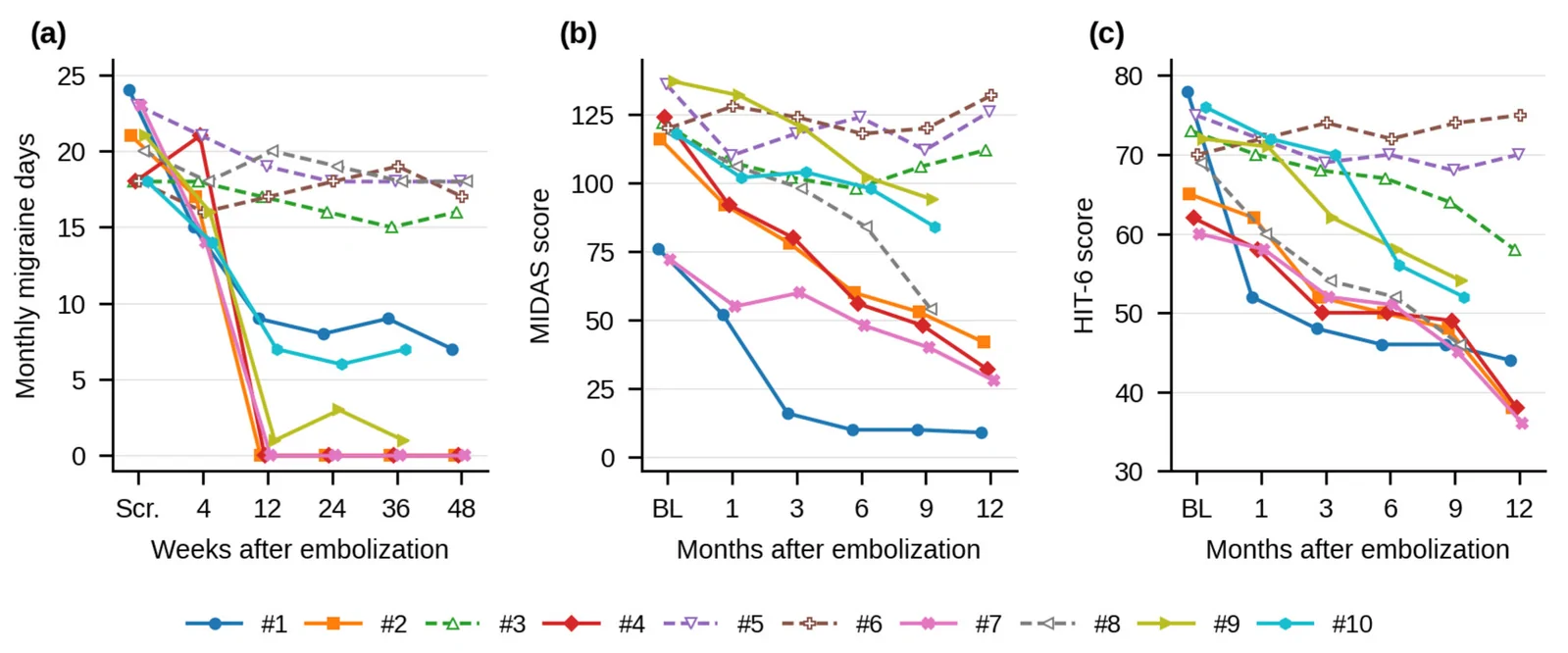
Individual trajectories of (**a**) migraine days per 28-day period from screening to week 48 (n = 10; n = 8 at week 48), (**b**) MIDAS scores and (**c**) HIT-6 scores from baseline to 12 months (n = 10; n = 7 at 12 months). Each line represents one patient. Solid lines and filled markers denote patients with a reduction in monthly migraine days of at least 50% at week 12 (patients 1, 2, 4, 7, 9, and 10); dashed lines and open markers denote patients without such a reduction (patients 3, 5, 6, and 8). Points are horizontally jittered to reduce overlap; patients 2, 4, and 7 had no migraine days from week 12 onwards. BL, baseline (pre-embolization); Scr., screening period.

Days with acute medication per 28-day period fell in parallel: median 20.0 (IQR 19.0–21.8) at baseline, 18.0 (16.2–20.0) at 4 weeks, 7.5 (0.2–16.8) at 12 weeks, 6.5 (0.8–16.8) at 24 weeks, 7.5 (0.0–15.8) at 36 weeks and 12.5 (0.0–17.2; n = 8) at 48 weeks (Table 3; per-patient values in Table S12). In the sensitivity analysis excluding patient 9 (previously implanted nerve stimulator), a reduction of at least 50% in monthly migraine days at 12 weeks was achieved by five of nine patients (55.6%; 95% CI 21.2–86.3), and the median change from baseline was −11 days (IQR −18 to −1); because patient 9 had not reached the 48-week period, the 48-week results are unchanged (Table S10). Individual trajectories of monthly migraine days, MIDAS and HIT-6 are shown in Figure 9.

The response was not immediate. At 4 weeks no patient had achieved a 50% reduction in migraine days (0 of 10; 95% CI 0–31), the median reduction was only −3 days, and days of non-migraine headache increased relative to baseline in eight of ten patients, consistent with post-embolization headache. At 8 weeks (days 29–56) the median number of monthly migraine days was 12.5 (IQR 10.2–16.5), the median change from baseline was −6.5 days (IQR −12.0 to −3.5), and a reduction of at least 50% was achieved by three of ten patients (30%; 95% CI 6.7–65.2; patients 1, 2, and 7), with five of ten reaching the 30% threshold. Separation from baseline appeared between 4 and 12 weeks and was maintained thereafter.

The apparent rise in median migraine days between 36 and 48 weeks reflects cohort composition rather than loss of effect: the two patients who had not yet reached the 48-week period (patients 9 and 10) were both responders. Restricted to the eight patients with 48-week data, the median was 12.0 days at 36 weeks and 11.5 days at 48 weeks.

Results did not differ appreciably between the two centers (three patients treated in Bishkek, seven in Saint Petersburg, all by the same operator): branch-level technical success was 11 of 12 target branches in Bishkek and 18 of 28 in Saint Petersburg, patient-level technical success two of three and two of seven, and a reduction in monthly migraine days of at least 50% at 12 weeks was achieved by two of three and four of seven patients. With three patients at one center, these figures are descriptive only.

Patients whose technical result was least complete did not respond: patient 5 (SPADES grade 0 in two branches and grade I–II in the remaining two) and patient 6 (grade 0 in one branch and grade II in two) showed reductions in monthly migraine days of 21.7% and 5.6% respectively. Conversely, a high grade in most branches did not guarantee a response: in patients 3 and 8, who achieved grade III or higher in three of four branches, monthly migraine days at 48 weeks had decreased by only 11.1% and 10.0%. At the patient level, all four patients with complete technical success (grade III or higher in all four branches; patients 1, 2, 9, and 10) achieved a reduction in monthly migraine days of at least 50% at 12 weeks, compared with two of the six patients with incomplete technical success (patients 4 and 7; descriptive, Fisher exact *p* = 0.076). Incomplete embolization therefore did not invariably mean treatment failure, but in this cohort it was associated with a lower response rate. This observation rests on four non-responders among ten patients, is unadjusted and unblinded, and is stated as a hypothesis for a controlled trial rather than as a finding.

These analyses are exploratory. They derive from an unblinded, uncontrolled cohort with incomplete follow-up and are not adjusted for multiplicity; they cannot be distinguished from a placebo response. The registered primary efficacy endpoint will be analyzed formally when enrolment is complete.

### 3.6. Dynamics of Anxiety and Depression on the HADS

**Figure 10:**
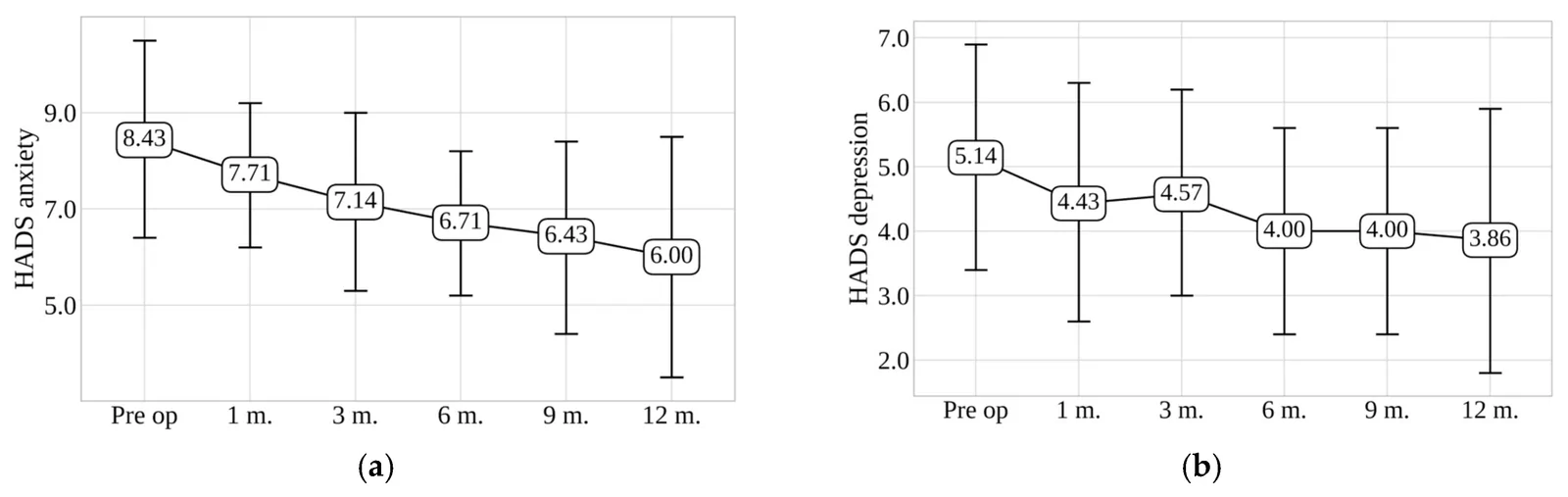
Dynamics of psychological outcomes assessed by the Hospital Anxiety and Depression Scale (HADS). (**a**) HADS anxiety scores from baseline (pre-operative) to 12 months. (**b**) HADS depression scores from baseline (pre-operative) to 12 months. Data are presented as means with 95% confidence intervals. n = 7 (patients with 12-month data); baseline values therefore differ from those in Table 1 (n = 10).

Anxiety scores decreased across the timepoints, from 8.4 ± 2.2 at baseline to 6.0 ± 2.7 (95% CI 3.5–8.5) at 12 months (Friedman test, *p* = 0.001; n = 7; Figure 10a). Depression scores did not change appreciably, from 5.1 ± 1.9 at baseline to 3.9 ± 2.2 at 12 months (*p* = 0.318; n = 7; Figure 10b). Intermediate values are given in Table S5. Baseline values in Section 3.6, Section 3.7 and Section 3.8 refer to the seven patients with 12-month data and therefore differ from the whole-cohort values in Table 1 (n = 10).

### 3.7. Dynamics of Disability Assessment on the MIDAS

**Figure 11:**
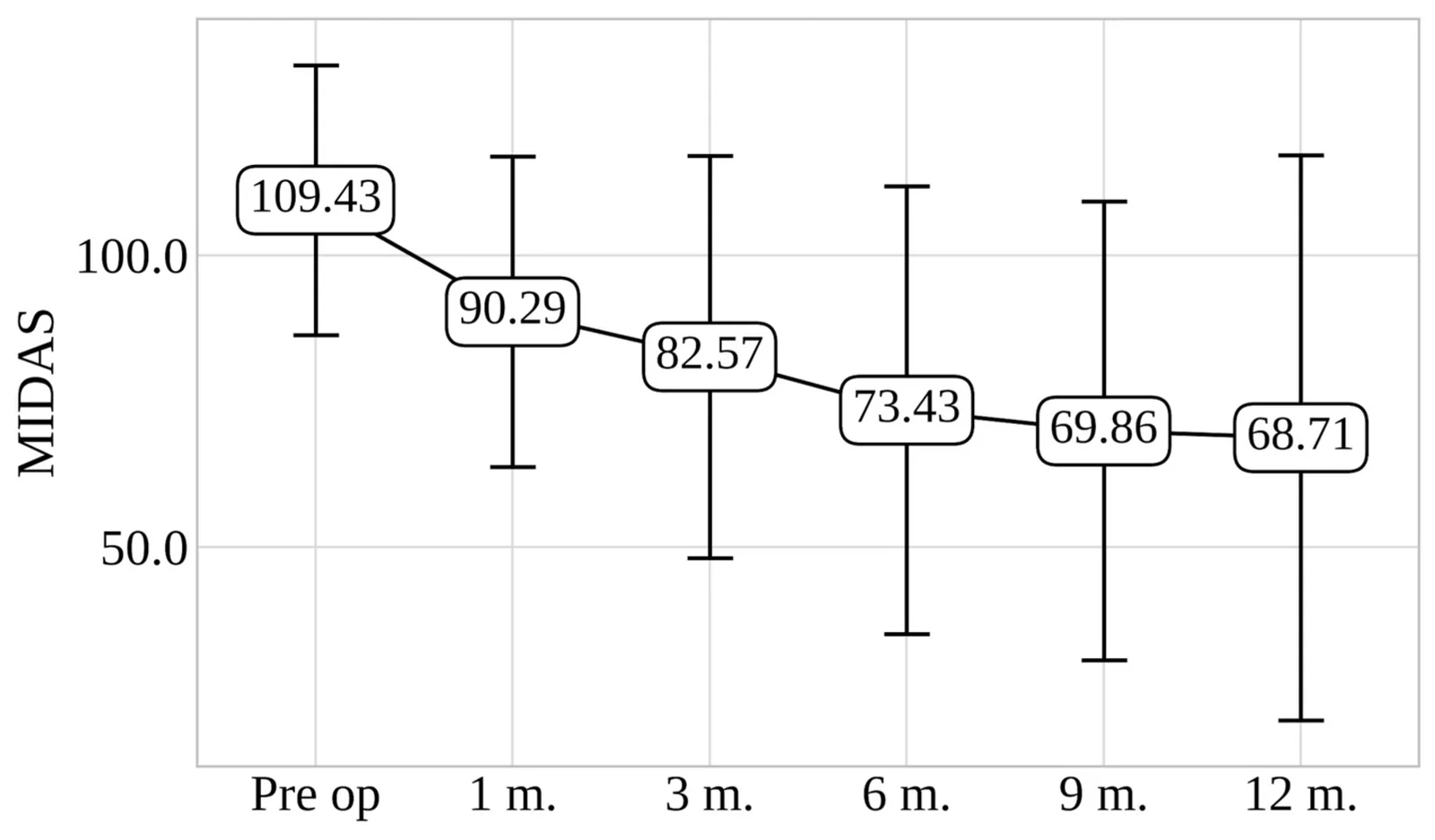
Dynamics of Migraine Disability Assessment (MIDAS) scores from baseline (pre-operative) to 12 months after embolization. Data are presented as means with 95% confidence intervals. n = 7 (patients with 12-month data); baseline values therefore differ from those in Table 1 (n = 10).

In the seven patients with complete 12-month data, the MIDAS score across the six scheduled timepoints decreased from 109.4 ± 25.0 at baseline to 68.7 ± 52.4 at 12 months (Friedman test, *p* < 0.001; Figure 11 and Table S6). This analysis is exploratory, uncontrolled, and unblinded; no causal attribution of the change to embolization is possible.

### 3.8. The Dynamics of Headache According to HIT 6

**Figure 12:**
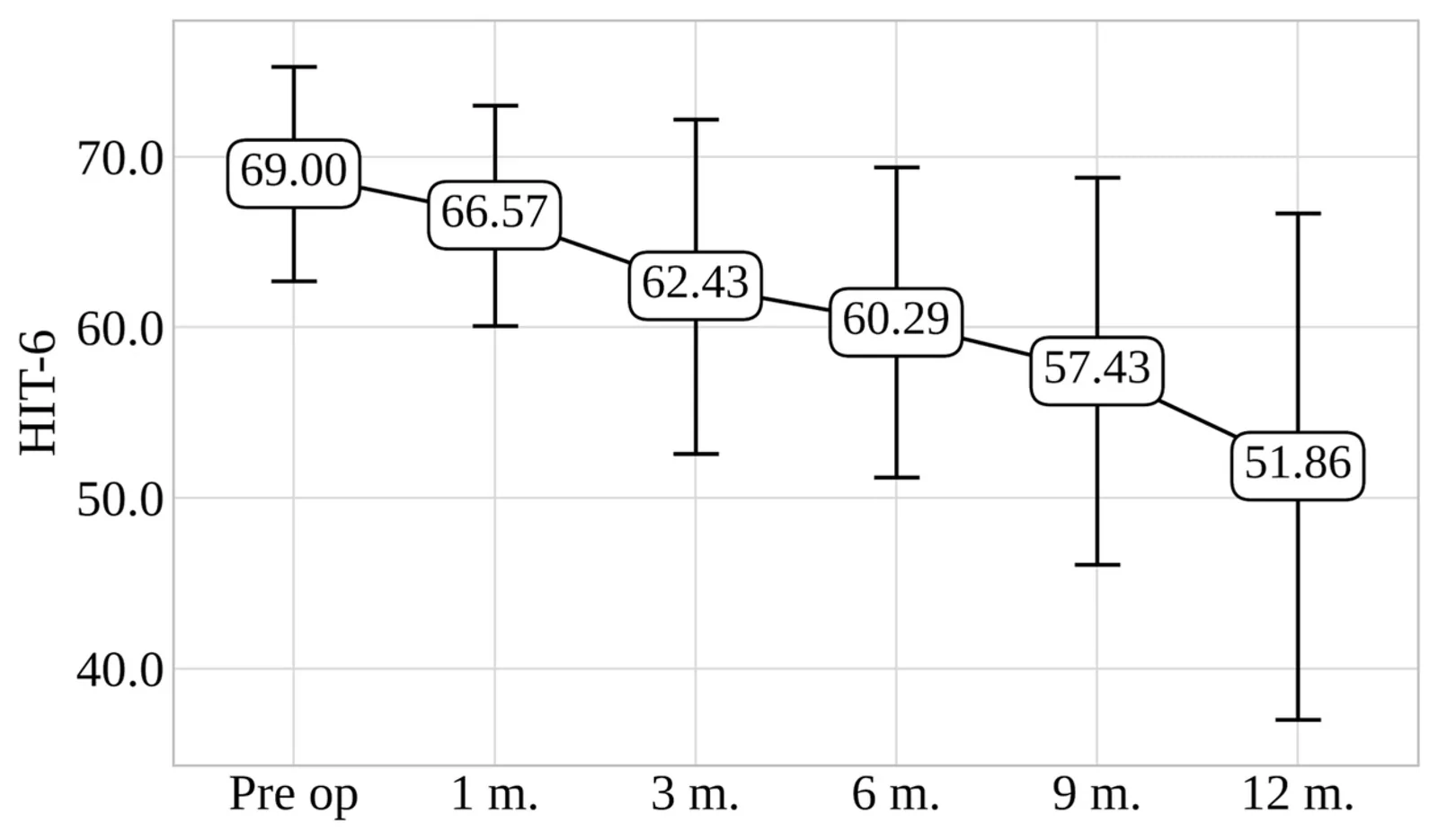
Dynamics of Headache Impact Test-6 (HIT-6) scores from baseline (pre-operative) to 12 months after embolization. Data are presented as means with 95% confidence intervals. n = 7 (patients with 12-month data); baseline values therefore differ from those in Table 1 (n = 10).

In the seven patients with 12-month data, the HIT-6 score decreased from 69.0 ± 6.8 at baseline to 51.9 ± 16.1 at 12 months (Figure 12), with a decline across the six scheduled timepoints (Friedman test, *p* < 0.001; n = 7; Table S7). This analysis is exploratory, uncontrolled, and unblinded).

### 3.9. Safety of the Methodology

No serious adverse events related to MMA embolization occurred in the 10 treated patients (0/10; 95% CI 0–30.8), and no intraoperative or early postoperative complication required additional treatment or prolonged hospitalization. Non-serious events attributable to the procedure were post-embolization headache and a headache attack provoked during follow-up angiography in two of the seven patients who underwent it (Section 3.4). Post-embolization headache was recorded in nine of ten patients (patient 6 had none). It began within the first 6 h after the procedure, on the day of embolization, lasted a median of 15 days (IQR 13–18; range 11–32, the longest in patient 1, extending beyond the first 28-day period), was bilateral. In the diary this was reflected as an increase in non-migraine headache days during the first 28 days relative to baseline in eight of ten patients. These headaches were classified as non-migraine on the basis of the prospective diary, which recorded headache characteristics and accompanying symptoms: days on which the headache did not meet ICHD-3 criteria for migraine were counted as non-migraine headache days.

Diffusion-weighted MRI within 48 h after the procedure showed no new ischaemic lesion in any patient. Ophthalmological examination before discharge showed no change from baseline in visual acuity, fundus, or visual fields in any patient. No cranial nerve deficit (including facial nerve palsy), no scalp or skin change (alopecia, necrosis), and no access-site complication was observed at discharge or at any follow-up visit. No venous complication was identified: on the venous phase of follow-up angiography the dural venous sinuses and the venous structures reached by the embolic agent were patent in all seven patients examined (Section 3.4, Table S14); MR venography was not performed.

On the modified Rankin Scale (mRS), all patients scored 0 at every assessment: at discharge and at 4, 12, 24, and 36 weeks in all ten patients, and at 48 weeks in the eight patients who had reached that visit.

The absence of serious events in 10 patients is compatible with a true rate of serious complications of up to 30.8%; these data demonstrate initial procedural feasibility but do not establish safety.

## 4. Discussion

In this first series, bilateral embolization of both MMA branches with non-adhesive liquid embolic agents was technically feasible, although SPADES grade III or higher in all four target branches was achieved in only four of ten patients, and the accompanying clinical changes were substantial but uncontrolled. These data demonstrate initial procedural feasibility; they do not establish safety or efficacy.

The clinical effect was assessed by a reduction in the frequency of migraine days of ≥50 percent, which was achieved in six out of ten patients by week 12 and in four out of eight patients by week 48. A comparable proportion of responders has been reported by Fakih et al. (2025) [18] after intra-arterial lidocaine infusion into the MMA, although with a different outcome measure: five5 of eight patients (62.5%) achieved a reduction of at least 50% in MIDAS by the third month (MIDAS 86.3 ± 40.1 → 23.5 ± 26.8; *p* = 0.001). However, in absolute terms, our effect developed more slowly: over the first three months, the MIDAS score in our cohort decreased by only 24.5% (109.4 ± 25.0 → 82.6 ± 37.2), whereas in Fakih et al. [18], it fell by 72.8% over the same period. This is consistent with our own diary data, in which no patient had achieved a 50% reduction in migraine days by week 4 (0 of 10; 95% CI 0–31). It is possible that this difference reflects variations in underlying mechanisms: the temporary chemical blockade of nociception with lidocaine acts rapidly, while the effect of permanent devascularization following embolization develops over a period of weeks.

This delay also contrasts with the course after MMA occlusion with coils or cyanoacrylate, where improvement was early: after unilateral coil occlusion, headache decreased within 24–48 h in 12 of 15 patients and worsened in none [20], and after cyanoacrylate occlusion headache-related disability had already improved by the 30-day visit [21]. Several factors may contribute: (i) post-embolization headache, which increased non-migraine headache days in eight of ten patients during the first 28 days and may have coexisted with, or complicated the classification of, migraine days—Xu et al. chose coils specifically to avoid the postoperative headache that may follow liquid embolic agents [20]; (ii) a local inflammatory reaction of the dura to DMSO and the ethylene–vinyl alcohol copolymer, which is probably more pronounced with deep penetration of a non-adhesive agent to the capillary level than with proximal occlusion by coils or glue; and (iii) a gradual process such as remodeling of dural innervation, if that mechanism operates. At 8 weeks, three of ten patients had reached a 50% reduction in migraine days, so that the response accrued between weeks 4 and 12 rather than being absent and then abrupt. Whether this delay is specific to non-adhesive agents requires evaluation in a controlled trial.

Vanzin and Manzato [21] performed bilateral MMA occlusion with cyanoacrylate in 10 patients with refractory chronic migraine selected by a positive intra-arterial lidocaine test; postoperative headache exacerbation occurred within the first 6 h and resolved completely before discharge within 24 h, and HIT-6 had improved by 30.5 points at 6 months without neurological or technical complications. In our cohort post-embolization headache lasted considerably longer—a median of 15 days (range 11–32) in nine of ten patients—which may reflect the larger volume and deeper dural penetration of a DMSO-based agent; a further difference between the series is the lidocaine-test selection in that study.

In the study by Xu et al. (2026) [20] which included 15 patients who underwent coil embolization, there was a significant improvement in both the MIDAS and HIT-6 scores (Z = −3.077, *p* = 0.002, and t = 4.730, *p* < 0.001, respectively). The individual values given in Table 2 and Table 3 of that publication show that monthly headache days at 3 months had decreased from a median of 23 (IQR 11–30) to 5 (IQR 1–10), MIDAS from a median of 26 to 11 and HIT-6 from a mean of 63.7 to 50.6, and that a reduction in headache days of at least 50% was achieved by 10 of 15 patients (66.7%); in our cohort a reduction in monthly migraine days of at least 50% was achieved by 60% of patients at 12 weeks and by 50% at 48 weeks. The endpoints are not identical, however: Xu et al. [20] counted all headache days after unilateral coil occlusion of the MMA trunk, whereas we counted migraine days after bilateral embolization of both branches. The two series therefore agree in direction, but neither is controlled, and agreement between two uncontrolled series does not by itself support a treatment effect.

A direct quantitative comparison of our safety data with those of the large randomized trials of MMA embolization for chronic subdural haematoma is limited for several reasons, of which the first is the difference in patient populations. Specifically, elderly patients with cSDH were compared with younger, middle-aged patients afflicted with migraine. In cases of cSDH, an increase in the diameter of the MMA and the presence of well-developed neoangiogenic capillary networks are observed [33,34]. In the context of migraine, the anatomical changes in the MMA remain to be elucidated. Additionally, the objectives of these procedures are not uniform. However, large randomized clinical trials of MMA embolization for cSDH establish a benchmark for the complications to be expected from MMA embolization in general.

In the study by Fiorella et al. (2025) (STEM) [13], the incidence of major disabling stroke or death within 30 days was three percent in both the embolization group and the control group (four out of one hundred and forty-four and five out of one hundred and sixty-six, respectively). In the study by Davies et al. (2024) (EMBOLISE) [14], serious adverse events associated with the embolization procedure occurred in four out of one hundred and ninety-seven patients (2.0%) by day 30, including a disabling stroke in two patients (1.0%); the risk of recurrence or progression of the hematoma requiring reoperation was 4.1% compared with 11.3% in the control group (RR 0.36; 95% CI 0.11–0.80; *p* = 0.008). The absence of significant adverse events in our series of 10 patients is consistent with the low (single-digit percentage) incidence of serious complications of MMA embolization in these significantly larger studies. However, the sample size of our study is too small to formally confirm a comparable level of safety.

The study by Xu et al. [20], also reported two adverse events in 15 patients (13.3 per cent: hematoma at the puncture site and transient exacerbation of chronic back pain), which were not directly related to embolization. This outcome is analogous to the absence of significant adverse events in our series (0 out of 10; 95% CI 0–30.8), although the limited sample sizes of both groups preclude a formal comparison of complication rates.

The incidence of anastomoses with the ophthalmic artery in our series (73.7 per cent; 14 out of 19 MMA cases available for assessment) is considerably higher than the figures reported in published angiographic studies of patients with cSDH. In particular, Shotar et al. (2022) [35] reported a frequency of 19 per cent (26 out of 140 MMA), while Pilawska et al. (2024) [36] reported only 3.31 per cent (four out of one hundred and twenty-one). The most likely explanation is methodological: anastomoses were sought deliberately by superselective angiography of each branch, whereas Shotar et al. [35] recorded an angiographic relationship with the ophthalmic artery on standard diagnostic DSA. A contribution from population differences cannot be excluded, but a four- to twenty-fold difference in reported frequency is more readily explained by the method of detection than by the biology of migraine. Notwithstanding, the clinical risk associated with the high prevalence of ophthalmic anastomoses necessitates particular attention during MMA embolization [37,38].

However, anastomoses—especially with the ophthalmic artery (73.7% in our series)—complicate complete embolization, since proximal collaterals risk inadvertent embolism in the internal carotid artery territory. In some cases, in particular with MMA ectopia (coming from the ophthalmic artery), embolization has to be abandoned. With careful angiographic assessment of anastomoses and correct wedge positioning, no procedure-related neurological complication was observed in this small series (0 of 10; 95% CI 0–30.8), and headache-related disability decreased. The high frequency of ophthalmic anastomoses dictates the need for routine superselective angiography. Branch tortuosity and the Adachi configuration, rather than branch caliber alone, determined the technical result. Bilateral treatment was chosen because no patient had strictly unilateral pain; whether bilateral embolization is necessary cannot be established from this series, since only one patient was treated unilaterally.

The distribution of MMA types according to Adachi in our series (the parietal branch could not be catheterized in any of the four arteries of types three or four, whereas it was catheterized in 11 of the 16 arteries of other types) cannot be compared directly with published anatomical series, which report the frequency of each configuration rather than its consequences for catheterization. Shotar et al. (2022) [35] found dominance of the anterior branch in 57 of 140 MMAs (41%) using an extended Adachi classification, and Pilawska et al. (2024) [36] reported subtype IC as the most frequent (19.82%) and subtype IB as the least frequent (6.31%). Notably, Shotar et al. [35] reported only moderate interobserver agreement for this classification (kappa 0.53, 95% CI 0.39–0.67), which suggests that anatomical typing of the MMA is itself a source of variability and that our observation requires confirmation in a larger series.

Branch diameter was at the limit of what current microcatheters can be wedged into: the non-catheterized parietal branches had a median diameter of 0.8 mm and 0.7 mm, which overlaps with the range of the catheterized parietal branches, so that diameter alone does not explain why catheterization failed. The wedge technique or a balloon microcatheter was used in 24 of the 30 catheterized branches (80.0%) and was the preferred condition for safe delivery of a non-adhesive embolic agent, while using a combination of SQUID 12 (for primary distal penetration) and SQUID 18 (for creating a proximal plug) made it possible to consistently embolize both small arterial branches and the proximal part of the MMA.

The mechanism by which MMA embolization might reduce migraine frequency was not investigated in this study, and any explanation remains hypothetical. Because dilatation of the meningeal arteries is not a prerequisite for migraine pain [9,10], a purely haemodynamic explanation is unlikely. Consistent with current concepts of the trigeminovascular system [5,8,11,39,40,41], candidate mechanisms include chemical or ischaemic injury to perivascular trigeminal nociceptors by DMSO and the ethylene–vinyl alcohol copolymer, and aseptic inflammation of the dura with subsequent remodeling of its innervation. None of these mechanisms was measured, and the delayed onset of improvement (Section 3.5) is compatible with more than one of them.

Our report has limitations. First, given its single-arm design and the absence of blinding, we cannot exclude the placebo effect, and the magnitude of change in MIDAS and HIT-6 does not by itself distinguish a treatment effect from a placebo response: sham-controlled trials of interventional procedures for migraine have shown substantial improvement in the sham arms [42,43], and in a systematic review the proportion of responders after sham surgery was 58% (95% CI 37–77) [44]. Regression to the mean is also likely, because eligibility required a high number of headache days documented during a single 28-day screening period, whereas the natural course of chronic migraine is variable—nearly three quarters of patients with chronic migraine fall below the 15-day threshold at least once within a year [45], and about a quarter remit to episodic migraine over two years [46]. Because acute medication use fell in parallel with migraine days in the responders (Table S12), the contribution of reduced medication use to the observed changes cannot be separated from any effect of the procedure; however, no structured withdrawal was undertaken during the trial, and all enrolled patients had previously failed a documented withdrawal attempt. Second, this series is an early analysis with a very small sample size to confirm the feasibility of the treatment approach, thus no definitive conclusions can be drawn. Third, follow-up is of unequal duration: all ten patients have at least 9 months, eight have completed the 48-week diary period and seven have attended the 12-month visit. Fourth, SPADES is an investigator-developed scale that has not been externally validated, and technical success—the main outcome of this report—was defined post hoc on the basis of this scale; the interim analysis itself was not prespecified. Fifth, quality of life was not measured with a dedicated instrument. Sixth, patients were selected by different physicians at the two centers and all procedures were performed by a single operator, so the technical results may not be reproducible elsewhere. Seventh, the embolic agent extended into the dural veins in eight of ten patients; the long-term consequences of venous extension in young patients are unknown and require specific surveillance of the venous system. Eighth, one patient (patient 9) had a previously implanted nerve stimulator, which may confound the headache outcome (sensitivity analysis in Section 3.5 and Table S10). Ninth, in the two patients treated through a detachable-tip microcatheter a solid component remained within the embolic cast, so that their treatment was not purely liquid embolization. Tenth, clinical outcomes were collected by neurologists who had not performed the procedure but were aware that every patient had been treated; no independent third-party or blinded assessment was undertaken.

Further experience is needed to clarify whether complete MMA shutdown with a venous component independently predicts long-term response; until the safety of venous extension is established, it should be regarded as acceptable rather than as a technical goal. Overall, bilateral frontal and parietal MMA embolization achieving deep arterial penetration is technically in chronic resistant migraine; whether it is effective remains to be shown in a sham-controlled trial.

## 5. Conclusions

In this interim analysis of an ongoing prospective, single-arm, multicenter trial, bilateral MMA embolization with non-adhesive liquid embolic agents was technically feasible: SPADES grade III or higher was achieved in 29 of 40 target branches (72.5%) and in all four target branches in four of ten patients, and no procedure-related neurological complication occurred (0 of 10; 95% CI 0–30.8); although, a sample of this size cannot establish safety. Two anatomical factors were related to the technical result: branch tortuosity, to how distally the catheter could be advanced, and the Adachi configuration, to whether the parietal branch could be catheterized. Distal anastomoses and anastomoses with the ophthalmic artery were frequent, so superselective angiographic assessment before embolization is mandatory. The embolic agent reached the dural veins in eight of ten patients, and the safety of venous extension requires specific surveillance. The accompanying reduction in migraine days and headache-related disability was obtained without a control group or blinding, cannot be separated from a placebo response, and will be tested when enrolment is complete.

## Figures and Tables

**Table 1:** Baseline patient characteristics (n = 10).

Characteristic	Value
Age, years (Mean ± SD)	41.4 ± 13.7
Age, years, median (IQR); range	44.0 (29.5–45.0); 24–67
Female, n (%)	9 (90.0%)
Male, n (%)	1 (10.0%)
Height, cm, (Mean ± SD)	170.6 ± 5.0
Body weight, kg, (Mean ± SD)	70.3 ± 6.0
Body mass index, kg/m^2^, (Mean ± SD)	24.1 ± 1.4
Body mass index, kg/m^2^, median (IQR); range	24.1 (23.9–24.8); 21.2–26.1
Postmenopausal, n (%)	2 (20.0%)
Age at migraine onset, years, median (IQR); range	14 (7–18); 6–22
Duration of migraine since onset, years, median (IQR); range	26 (22–34); 6–61
Duration of chronic migraine, years, (Mean ± SD)	7.1 ± 2.33
Number of failed oral preventive classes, median (IQR); (range)	3 (3–4); 3–5
History of surgical treatment for migraine, n (%)	1 (10.0%)
MIDAS, total score (Mean ± SD)	113.9 ± 22.3
MIDAS, total score, median (IQR); range	119.0 (116.5–123.5); 72–137
HIT-6, total score (Mean ± SD)	70.0 ± 6.0
HIT-6, total score, median (IQR); range	71.0 (66.0–74.5); 60–78
HADS: anxiety (Mean ± SD)	8.4 ± 2.17
HADS: depression (Mean ± SD)	6.0 ± 2.1
Recruiting site: Saint Petersburg/Bishkek, n	7/3
Follow-up at data cut-off, months, median (IQR); range	18.5 (12.3–20.0); 9–22

Abbreviations: HIT-6, Headache Impact Test-6; MIDAS, Migraine Disability Assessment; HADS, Hospital Anxiety and Depression Scale; SD, standard deviation.

**Table 2:** Individual technical SPADES grades and clinical outcomes (MIDAS and HIT-6) for each patient.

Patient №	Sex	Age	SPADES				MIDAS			HIT-6			The Months Following the Operation up to the Final Clinical Follow-Up	Migraine Status at the Time of the Last Clinical Follow-Up
			Frontal Br. R. MMA	Parietal Br. R. MMA	Frontal Br. L. MMA	Parietal Br. L. MMA	Baseline	6-Month	12-Month	Baseline	6-Month	12-Month		
1	female	45	III	III	III+Vens	III+Vens	76	10	9	78	62	48	22	Episodic migraine
2	female	57	III+Vens	III	III	III	116	60	42	65	50	38	21	No migraine attacks for more than 15 months
3	female	44	III	0	III+Vens	III	122	98	112	73	67	58	20	The severity of the attacks has decreased, but not their frequency
4	female	44	II+Vens	III	IV+Vens	III	124	56	32	62	50	38	20	No migraine attacks for more than 12 months
5	female	28	II	I	0	0	136	124	126	75	70	70	19	No effect
6	female	45	II	II	III	0	120	118	132	70	72	75	18	No effect
7	male	29	III+Vens	III	IV+Vens	II	72	48	28	60	51	36	16	No migraine attacks for more than 9 months
8	female	24	III+Vens	III	III+Vens	II	118	84		69	52		11	The severity of the attacks has decreased, but not their frequency
9	female	67	III	III+Vens	III	III+Vens	137	102		72	52		9	Episodic migraine
10	female	31	IV+Vens	III	III+Vens	III	118	98		76	52		9	Episodic migraine

**Table 3:** Diary-derived headache outcomes.

Timepoint	n	MMD, Median (IQR)	Change in MMD, Median (IQR)	≥50% MMD, n (%)	≥30% MMD, n (%)	MHD, Median (IQR)	≥50% MHD, n (%)	Acute Medication Days, Median (IQR)
Baseline	10	20.5 (18–22.5)	—	—	—	26.5 (23.5–27.8)	—	20.0 (19.0–21.8)
4 weeks	10	16.5 (15.2–18)	−3 (−4.8 to −2)	0 (0)	2 (20)	23.5 (22.2–24.8)	0 (0)	18.0 (16.2–20.0)
12 weeks *	10	8 (0.2–17)	−13 (−19.5 to −1.8)	6 (60)	6 (60)	8.5 (0.2–19.8)	6 (60)	7.5 (0.2–16.8)
24 weeks	10	7 (0.8–17.5)	−14 (−18 to −2.8)	6 (60)	6 (60)	7.5 (0.8–18)	6 (60)	6.5 (0.8–16.8)
36 weeks	10	8 (0.2–17.2)	−13 (−19.5 to −3.5)	6 (60)	6 (60)	8.5 (0.5–17.2)	6 (60)	7.5 (0.0–15.8)
48 weeks	8	11.5 (0–17.2)	−11 (−18.8 to −2)	4 (50)	4 (50)	13.5 (0.8–18.2)	4 (50)	12.5 (0.0–17.2)

* Registered primary efficacy endpoint of the trial. MMD, monthly migraine days; MHD, monthly headache days (migraine plus non-migraine headache days); IQR, interquartile range. Percentages are of the number analyzed at each timepoint. Patients 9 and 10 had not reached the 48-week period at the data cut-off.

## Data Availability

The data supporting the findings of this interim analysis are contained within the article and the Supplementary Materials: individual technical grades for all 40 branches are given in Table 2, and individual diary-derived outcomes for all 10 patients in Table S9. The trial is ongoing. De-identified individual participant data underlying the published results, together with the study protocol and the statistical analysis plan, will be made available by the corresponding author (doctorpetrovandrey@gmail.com) on reasonable request from qualified investigators after enrolment is complete and the primary analysis has been published, for proposals whose scientific merit and feasibility have been assessed by the investigators and approved by the ethics committees of the participating centers. Requests will be considered for five years from the date of publication. Data that could identify individual participants will not be shared.

## Supplementary Materials

The following supporting information can be downloaded at https://www.mdpi.com/article/10.3390/jcm15208151/s1, Table S1: Full inclusion and exclusion criteria; Table S2: Anatomical and technical characteristics of the middle meningeal artery and its frontal and parietal branches (n = 40 branches); Table S3: Microcatheter outer diameter according to the degree of branch tortuosity; Table S4: Frequency and location of middle meningeal artery anastomoses; Table S5: Hospital Anxiety and Depression Scale scores at each follow-up timepoint (n = 7); Table S6: Migraine Disability Assessment (MIDAS) scores at each follow-up timepoint (n = 7); Table S7: Headache Impact Test-6 (HIT-6) scores at each follow-up timepoint (n = 7); Table S8: Level of catheter tip placement by branch and by degree of branch tortuosity; Table S9: Per-patient monthly migraine days and monthly headache days; Table S10: Sensitivity analysis of diary-derived outcomes at 12 and 48 weeks with and without patient 9; Table S11: Previous preventive treatment and preventive medication during follow-up, by patient; Table S12: Days with acute (symptomatic) medication per 28-day period, by patient; Table S13: SPADES grades assigned by the operator and by the second reader, and the consensus grade, for each target branch; Table S14: Venous structures reached by the embolic agent in the 14 branches with venous extension (8 patients); File S1: CONSORT checklist for the extension to randomized pilot and feasibility trials, adapted for a single-arm design.

## Abbreviations

The following abbreviations are used in this manuscript:

BMI	body mass index
CGRP	calcitonin gene-related peptide
CI	confidence interval
CONSORT	Consolidated Standards of Reporting Trials
CRM	chronic resistant migraine
CT	computed tomography
DM	dura mater
DMSO	dimethyl sulfoxide
DSA	digital subtraction angiography
DWI	diffusion-weighted imaging
EEG	electroencephalography
EHF	European Headache Federation
HADS	Hospital Anxiety and Depression Scale
HIT-6	Headache Impact Test-6
ICH GCP	International Council for Harmonization Good Clinical Practice
ICHD-3	International Classification of Headache Disorders, 3rd edition
IQR	interquartile range
MHD	monthly headache days
MIDAS	migraine disability assessment
MMA	middle meningeal artery
MMD	monthly migraine days
MRI	magnetic resonance imaging
mRS	modified Rankin Scale
NLEA	non-adhesive liquid embolic agent
NYHA	New York Heart Association
PACAP	pituitary adenylate cyclase-activating polypeptide
PREEMPT	Phase III Research Evaluating Migraine Prophylaxis Therapy
SD	standard deviation
SPADES	Saint Petersburg Arterial Dural Embolization Scale
TVS	trigeminovascular system
