# Supplementary material for: Middle Meningeal Artery Embolization Using Non-Adhesive Liquid Embolic Agents for Chronic Resistant Migraine: Interim Feasibility and Safety Analysis of a Prospective Single-Arm Trial
Source: J Clin Med. 2026 Oct 20;15(20):8151. doi: 10.3390/jcm15208151 (PMC13706369; doi:10.3390/jcm15208151)
Supplement: Supplementary file 1 [file jcm-15-08151-s001.zip › File S1.pdf]

Supplementary Materials

Middle Meningeal Artery Embolization Using Non-Adhesive Liquid Embolic Agents for Chronic Resistant Migraine: Interim Feasibility and Safety Analysis of a Prospective Single-Arm Trial

Andrey Petrov, Ruslan Sharshebaev, Gulmira Biialieva, Sergei Ermakov, Arkady Ivanov, Ekaterina Samorukova, Anna Petrova, Julia Suvorova and Stalbek Akhunbaev

This file contains Tables S1–S14. The CONSORT checklist for the extension to randomised pilot and feasibility trials, adapted for a single-arm design, is provided separately as File S1; the study protocol is available from the corresponding author on request (see Data Availability Statement). All data are for the first 10 patients treated in the CHROME trial (ClinicalTrials.gov NCT06848166), with a data cut-off of 5 September 2026.

Table S1. Full inclusion and exclusion criteria

**Inclusion criteria — all of the following were required**	
**Criterion**	**Definition**
Written consent	The patient has read, understood, signed and dated the patient information sheet and the informed consent form prior to any study procedure.
Age	Men and women aged 21 to 70 years inclusive at the time of signing the informed consent form.
Diagnosis	Chronic migraine according to the International Classification of Headache Disorders, 3rd edition (2018), established at least 12 months before screening.
Resistance to treatment	Documented treatment-resistant migraine according to the European Headache Federation consensus criteria (2020) for at least 6 months before screening, that is, failure of at least three classes of preventive medication together with failure of non-pharmacological preventive approaches.
Attack frequency during screening	At least 14 headache days during the 28-day screening period, of which at least 8 met the criteria for migraine, documented prospectively in the daily headache diary.
Pain intensity	Maximum headache intensity on migraine days of at least 5 points on an 11-point numerical rating scale during the screening period.
Failure of botulinum toxin	Absence of a clinically meaningful response in headache frequency or intensity to a previous course of botulinum toxin type A (onabotulinumtoxinA or Relatox, 155–195 U every 12 weeks) administered according to the PREEMPT protocol.
Contraception	Willingness of men and women of reproductive age to use two effective methods of contraception simultaneously and to refrain from gamete donation from consent until the end of follow-up.

**Exclusion criteria — any of the following precluded participation**	
**Criterion**	**Definition**
Contraindications to the procedure	Any general or specific contraindication to endovascular embolization of the middle meningeal artery identified at screening by physical, laboratory or imaging examination.
Contrast allergy	Known allergy or intolerance to iodinated contrast media.

Exclusion criteria — any of the following precluded participation	
**Criterion**	**Definition**
Vascular and structural CNS abnormalities	Cerebral vascular malformations, arteriovenous malformations, intracranial aneurysms, brain tumour, decompensated hydrocephalus, arachnoiditis, disorders of cerebrospinal fluid circulation, craniovertebral junction anomalies including platybasia and clivus deformity, or haemodynamically significant stenosis or occlusion of the precerebral or cerebral arteries.
Head injury and previous surgery	Skull base fracture or its sequelae, and sequelae of previous neurosurgical procedures.
Psychiatric status	Diagnosed mental disorder, including clinically significant depression confirmed by a specialist at screening on the basis of the HADS and Hamilton scales, judged likely to distort the patient's assessment of their own condition or to compromise the safety of participation.
Endocrine disorders	Uncontrolled diabetes mellitus, hyperthyroidism or other serious endocrine disorder that could affect interpretation of efficacy and safety.
Substance dependence	Alcohol, drug or medication dependence.
Medication-overuse headache	Medication-overuse headache at screening or within the preceding 3 months [a].
Other headache disorders	Other chronic headache disorders of less than 12 months' duration, including trigeminal autonomic cephalalgias, new daily persistent headache, painful cranial neuropathies, retinal migraine, hemiplegic migraine, migraine with brainstem aura, idiopathic intracranial hypertension without papilloedema, and any secondary headache.
Differentiation of pain	Inability of the patient to distinguish migraine attacks from other headache types.
Migraine complications	Status migrainosus, persistent aura without infarction, migrainous infarction, or migraine aura-triggered seizure.
Body mass index	Body mass index below 18.5 or above 39.9 kg/m^2.
Late onset	First appearance of migraine after the age of 50 years.
Physical status	Metabolic syndrome or cachexia of any aetiology.
Severe cardiovascular disease	NYHA class III–IV heart failure, clinically significant ventricular arrhythmia, haemodynamically significant valvular disease, uncontrolled hypertension above 180/110 mmHg, or previous pulmonary embolism or deep vein thrombosis.
Lower-limb vascular disease	Clinically evident varicose veins or documented previous venous thrombosis.
Coronary artery disease	Ischaemic heart disease or severe or progressive coronary atherosclerosis.
Cerebrovascular disease	Stenosing atherosclerosis of the brachiocephalic arteries, previous stroke or transient ischaemic attack, or their sequelae.
Pregnancy	Confirmed pregnancy, breastfeeding, or planned pregnancy within 3 months.
Arrhythmia and cardiac surgery	Atrial fibrillation, patent foramen ovale, atrial septal defect, or any previous open-heart or coronary surgery.
Coagulopathy	Laboratory or clinical evidence of a coagulation disorder, including antiphospholipid syndrome.
Antithrombotic therapy	Anticoagulant or antiplatelet treatment that cannot be safely discontinued at least 5 days before the procedure.
Respiratory status	Uncontrolled asthma or severe chronic obstructive pulmonary disease.
Renal dysfunction	History of renal failure or estimated glomerular filtration rate below 60 mL/min with abnormal serum creatinine at screening.

Exclusion criteria — any of the following precluded participation	
**Criterion**	**Definition**
Hepatic dysfunction	History of hepatic failure or marked isolated elevation of transaminases at screening.
Chronic pain syndromes	Generalised pain syndrome or confirmed fibromyalgia.
Specific risks for women	Use of combined oral contraceptives with a documented history of thromboembolic complications of any location.
Neurological comorbidity	Epilepsy, previous epileptic syndromes or seizures, or any organic disorder of the nervous system other than migraine.
Blood-borne infection	History of, or positive serology at screening for, HIV, hepatitis B, hepatitis C or syphilis.
Malignancy	Malignant tumour diagnosed or treated within 5 years before screening, or current suspicion of active malignancy.
Other unstable conditions	Any other serious or unstable physical or psychological condition that in the investigator's opinion would preclude safe participation or bias the efficacy assessment.
Other trials	Concurrent participation in another interventional trial, or completion of such participation less than 30 days before screening.
Compliance	Inability to read or write, or unwillingness or cognitive inability to adhere to protocol procedures including daily diary completion.
Conflict of interest	Direct professional, administrative or family relationship between the patient and staff of the participating centre or the sponsor.

Criteria are reproduced from protocol version 1.0 of 1 October 2024, under which all 10 patients reported in this interim analysis were enrolled. [a] Operationalised as headache responsive to a documented withdrawal attempt undertaken 3–4 months before screening; see Section 2.2.2 (Headache-Specific Exclusions) of the main text.

Table S2. Anatomical and technical characteristics of the middle meningeal artery and its frontal and parietal branches (n = 40 branches)

Parameter	Category	Denominator	n	%	95% CI
Kinking of the frontal and parietal branches	Grade I	40 branches	4	10.0	2.8–23.7
	Grade II		5	12.5	4.2–26.8
	Grade III		8	20.0	9.1–35.6
	Normal		23	57.5	40.9–73.0
MMA configuration according to Adachi [a]	Type 1	40 branches	10	25.0	12.7–41.2
	Type 2a		14	35.0	20.6–51.7
	Type 2b		6	15.0	5.7–29.8
	Type 3		6	15.0	5.7–29.8
	Type 4		2	5.0	0.6–16.9
	Other		2	5.0	0.6–16.9
Wedge technique [b]	Yes	40 branches	21	52.5	36.1–68.5
	No		7	17.5	7.3–32.8
	Other		12	30.0	16.6–46.5
Reflux on microangiography after catheterisation [c]	Present	40 branches	7	17.5	7.3–32.8
	Absent		22	55.0	38.5–70.7
	Not applicable		11	27.5	14.6–43.9
Penetration into dural veins on the first SQUID injection [c]	Yes	40 branches	5	12.5	4.2–26.8
	No		30	75.0	58.8–87.3
	Not applicable		5	12.5	4.2–26.8
SQUID 12 used	Yes	40 branches	24	60.0	43.3–75.1
	No		16	40.0	24.9–56.7
SQUID 18 used	Yes	40 branches	26	65.0	48.3–79.4
	No		14	35.0	20.6–51.7

Percentages are of the stated denominator; 95% confidence intervals are exact (Clopper–Pearson).

[a] The Adachi type is a property of the artery rather than of the individual branch: both branches of one middle meningeal artery share the same type, so each of the 20 arteries contributes two branches. The corresponding artery-level counts are type 1, 5 arteries; type 2a, 7; type 2b, 3; type 3, 3; type 4, 1; other, 1.

[b] “Other” denotes branches in which delivery was achieved with a dual-lumen balloon microcatheter or by retrograde filling from an adjacent branch, so that the wedge technique was not applicable.

[c] “Not applicable” denotes branches in which the assessment could not be made because the branch was not catheterised or not embolised.

Table S3. Microcatheter outer diameter according to the degree of branch tortuosity

Device	Grade I (n = 3)	Grade II (n = 5)	Grade III (n = 6)	No tortuosity (n = 16)	All (n = 30)
1.5 Fr microcatheter	0 (0.0)	0 (0.0)	1 (16.7)	1 (6.3)	2 (6.7)
1.7 Fr microcatheter	0 (0.0)	0 (0.0)	1 (16.7)	9 (56.3)	10 (33.3)
2.1 Fr microcatheter	1 (33.3)	4 (80.0)	0 (0.0)	4 (25.0)	9 (30.0)
2.7 Fr microcatheter	2 (66.7)	1 (20.0)	0 (0.0)	2 (12.5)	5 (16.7)
Balloon microcatheter	0 (0.0)	0 (0.0)	4 (66.7)	0 (0.0)	4 (13.3)
**All branches**	3	5	6	16	30

Data are n (%) of the branches in each tortuosity category, for the 30 branches that were directly catheterised (frontal and parietal branches combined). Branches are clustered within patients (four branches per patient), so no test that treats branches as independent observations is reported; in a permutation test that preserves this clustering, the association of grade II–III tortuosity with the use of a balloon or a 1.5–1.7 Fr microcatheter (6 of 11 branches versus 10 of 19 with normal or grade I tortuosity) gives $p = 0.084$.

Table S4. Frequency and location of middle meningeal artery anastomoses

Location	Denominator	n	%	95% CI
Anastomosis of any location	20 arteries	17	85.0	62.1–96.8
Proximal anastomosis	20 arteries	10	50.0	27.2–72.8
Distal anastomosis	20 arteries	14	70.0	45.7–88.1
Anastomosis with the ophthalmic artery	19 arteries [a]	14	73.7	48.8–90.9

Anastomoses were assessed preoperatively by selective cerebral angiography in all 10 patients (20 arteries). Percentages are of the stated denominator; 95% confidence intervals are exact (Clopper–Pearson).

[a] In one artery the middle meningeal artery arose directly from the ophthalmic artery, so the presence of an anastomosis between the two could not be assessed separately.

Table S5. Hospital Anxiety and Depression Scale scores at each follow-up timepoint (n = 7)

**Anxiety subscale**		
**Timepoint**	**Mean ± SD**	**95% CI**
Baseline (pre-operative)	8.4 ± 2.2	6.4–10.5
1 month	7.7 ± 1.6	6.2–9.2
3 months	7.1 ± 2.0	5.3–9.0
6 months	6.7 ± 1.6	5.2–8.2
9 months	6.4 ± 2.2	4.4–8.4
12 months	6.0 ± 2.7	3.5–8.5
**Depression subscale**		
Timepoint	Mean ± SD	95% CI
Baseline (pre-operative)	5.1 ± 1.9	3.4–6.9
1 month	4.4 ± 2.0	2.6–6.3
3 months	4.6 ± 1.7	3.0–6.2
6 months	4.0 ± 1.7	2.4–5.6
9 months	4.0 ± 1.7	2.4–5.6
12 months	3.9 ± 2.2	1.8–5.9

Comparison across the six timepoints by the Friedman test: $p = 0.001$ for the anxiety subscale and $p = 0.318$ for the depression subscale. Values are for the seven patients who had reached the 12-month visit at the data cut-off of 5 September 2026 and therefore differ from the baseline values in Table 1 of the main text, which are for all 10 treated patients. The analysis is exploratory, uncontrolled and unblinded.

Table S6. Migraine Disability Assessment (MIDAS) scores at each follow-up timepoint (n = 7)

Timepoint	Mean ± SD	95% CI
Baseline (pre-operative)	109.4 ± 25.0	86.3–132.6
1 month	90.3 ± 28.7	63.7–116.9
3 months	82.6 ± 37.2	48.1–117.0
6 months	73.4 ± 41.4	35.1–111.8
9 months	69.9 ± 42.5	30.6–109.2
12 months	68.7 ± 52.4	20.3–117.1

All values are for the seven patients who had reached the 12-month visit at the data cut-off of 5 September 2026. Comparison across the six timepoints by the Friedman test: $p < 0.001$. The analysis is exploratory, uncontrolled and unblinded; no causal attribution to the procedure is possible. Baseline values differ from Table 1 of the main text, which reports all 10 treated patients.

Table S7. Headache Impact Test-6 (HIT-6) scores at each follow-up timepoint (n = 7)

Timepoint	Mean ± SD	95% CI
Baseline (pre-operative)	69.0 ± 6.8	62.7–75.3
1 month	66.6 ± 7.0	60.1–73.0
3 months	62.4 ± 10.6	52.6–72.2
6 months	60.3 ± 9.8	51.2–69.4
9 months	57.4 ± 12.3	46.1–68.8
12 months	51.9 ± 16.1	37.0–66.7

All values are for the seven patients who had reached the 12-month visit at the data cut-off of 5 September 2026. Comparison across the six timepoints by the Friedman test: $p < 0.001$. The analysis is exploratory, uncontrolled and unblinded; no causal attribution to the procedure is possible. Baseline values differ from Table 1 of the main text, which reports all 10 treated patients.

Table S8. Level of catheter tip placement

By branch

Level of tip placement	Frontal branches (n = 19)	Parietal branches (n = 11)	All branches (n = 30)
Level I (proximal)	5 (26.3)	3 (27.3)	8 (26.7)
Level II (medial)	13 (68.4)	3 (27.3)	16 (53.3)
Level III (parasagittal)	1 (5.3)	5 (45.5)	6 (20.0)
**All catheterised branches**	19	11	30

Data are n (%) of the catheterised branches in each column. Branches are clustered within patients, so the difference in distribution between frontal and parietal branches is reported descriptively.

By degree of branch tortuosity

Tortuosity	Level I	Level II	Level III	All
No tortuosity	2	9	5	16
Grade I	0	2	1	3
Grade II	2	3	0	5
Grade III	4	2	0	6
**All**	8	16	6	30

No branch with grade II or III tortuosity was catheterised beyond Level II, and 6 of these 11 branches were catheterised only to Level I, whereas all 6 branches reaching Level III had normal or grade I tortuosity. Spearman $\rho = -0.50$ (95% CI −0.82 to −0.07 by bootstrap resampling of patients, 10 000 replicates); in a permutation test that preserves the clustering of branches within patients, $p = 0.058$. Branch diameter was not associated with the level reached ($\rho = 0.18$).

Table S9. Per-patient monthly migraine days and monthly headache days

Patient	MMD baseline	MMD 4 wk	MMD 8 wk	MMD 12 wk	MMD 24 wk	MMD 36 wk	MMD 48 wk	MHD baseline	MHD 4 wk	MHD 8 wk	MHD 12 wk	MHD 24 wk	MHD 36 wk	MHD 48 wk
1	24	15	11	9	8	9	7	28	24	12	10	9	10	9
2	21	17	8	0	0	0	0	23	22	9	0	0	0	0
3	18	18	21	17	16	15	16	22	23	26	24	18	15	18
4	18	21	13	0	0	0	0	20	25	15	0	0	0	1
5	23	21	18	19	18	18	18	24	28	23	25	25	20	25
6	18	16	15	17	18	19	17	26	25	20	19	18	20	19
7	23	14	7	0	0	0	0	25	18	9	0	0	0	0
8	20	18	17	20	19	18	18	28	26	20	20	20	18	18
9	21	16	12	1	3	1	—	27	23	14	1	3	2	—
10	18	14	10	7	6	7	—	27	20	11	7	6	7	—

MMD, monthly migraine days; MHD, monthly headache days (migraine days plus days of non-migraine headache). Values are counts within consecutive 28-day periods anchored to the day of the procedure: days 1–28 (4 weeks), 29–56 (8 weeks), 57–84 (12 weeks), 141–168 (24 weeks), 225–252 (36 weeks) and 309–336 (48 weeks); baseline is the 28-day screening period. A migraine day was defined according to the International Classification of Headache Disorders, 3rd edition. Em dash denotes a period not yet reached at the data cut-off of 5 September 2026. No data were imputed.

Table S10. Sensitivity analysis of diary-derived outcomes at 12 and 48 weeks with and without patient 9

Analysis set	n	MMD, median (IQR)	Change in MMD from baseline, median (IQR)	≥50% reduction in MMD, n (%)	≥30% reduction in MMD, n (%)	MHD, median (IQR)
**12 weeks**						
All patients	10	8.0 (0.2–17.0)	−13.0 (−19.5 to −1.8)	6 (60)	6 (60)	8.5 (0.2–19.8)
Excluding patient 9	9	9.0 (0.0–17.0)	−11.0 (−18.0 to −1.0)	5 (56)	5 (56)	10.0 (0.0–20.0)
**48 weeks**						
All patients	8	11.5 (0.0–17.2)	−11.0 (−18.8 to −2.0)	4 (50)	4 (50)	13.5 (0.8–18.2)
Excluding patient 9	8	11.5 (0.0–17.2)	−11.0 (−18.8 to −2.0)	4 (50)	4 (50)	13.5 (0.8–18.2)

Patient 9 had a previously implanted nerve stimulator for migraine (see Section 2.2.2, Neurological Exclusions, of the main text). Values are computed from the per-patient data in Table S9; medians and interquartile ranges (IQR) are given for monthly migraine days (MMD) and monthly headache days (MHD), and responder counts are of the number analysed at each timepoint. The 48-week rows are identical because patient 9 had not reached the 48-week period at the data cut-off of 5 September 2026. The analysis is exploratory, uncontrolled and unblinded.

Table S11. Previous preventive treatment and preventive medication during follow-up, by patient

Patient	Failed oral preventive classes before enrolment [a]	Botulinum toxin A before enrolment	Anti-CGRP monoclonal antibody before enrolment	Preventive medication at enrolment (continued unchanged)	New preventive medication started during the 48-week follow-up
1	Beta-blocker (propranolol); topiramate; tricyclic antidepressant (amitriptyline)	2 cycles (2022–2023), no response	Erenumab, 6 months, no response; fremanezumab, single dose, discontinued (intolerance)	Venlafaxine; pregabalin	Trazodone (for insomnia) [b]
2	Not documented in detail [c]	4 cycles, no response	None	Not documented	None
3	SNRI (venlafaxine); topiramate; tricyclic antidepressant (amitriptyline); flunarizine	4 cycles (2023), <30% reduction	None (not reimbursed)	Venlafaxine; topiramate	None
4	Topiramate; SNRI (venlafaxine, duloxetine); beta-blocker (propranolol)	2 cycles (2022), no response	Erenumab, 10/2020–05/2022; relapse after discontinuation	None	None
5	Beta-blocker (propranolol); valproate; tricyclic antidepressant (amitriptyline); SNRI (venlafaxine); flunarizine	6 cycles (2021–2022), waning effect	Fremanezumab, 12 months (2023), loss of effect	None	None
6	Topiramate; beta-blocker (propranolol); tricyclic antidepressant (amitriptyline)	2 cycles (2023), no response	None	Topiramate	None
7	Beta-blocker (metoprolol); valproate; tricyclic antidepressant (amitriptyline); gabapentin [d]	3 cycles (2023), <30% reduction	None	None	None
8	Topiramate; tricyclic antidepressant (amitriptyline); flunarizine; candesartan; SNRI (venlafaxine)	2 cycles, no response	None (not started: Raynaud's phenomenon)	None	None
9	Beta-blocker (propranolol); topiramate; tricyclic antidepressant (amitriptyline); other antidepressants and anticonvulsants (not documented in detail)	Yes, temporary relief only (details not documented)	Erenumab, 5 months (2023–2024), stopped for constipation	None	None
10	Candesartan; topiramate; tricyclic antidepressant (amitriptyline); pregabalin [d]; other antidepressants (not documented in detail)	3 cycles, no response	None	Pregabalin	None

Classes are those listed in the European Headache Federation consensus statement on resistant and refractory migraine (2020). All 10 patients had failed at least three classes of oral preventive medication and at least one course of botulinum toxin type A; gepants were not available at either centre. Non-pharmacological preventive approaches (aerobic exercise, cognitive behavioural therapy, relaxation or biofeedback training, sleep hygiene) had been tried by all patients. Patient 9 had an occipital nerve stimulator implanted in 2014 (Section 2.2.2, Neurological Exclusions).

[a] Doses, durations and reasons for failure (lack of efficacy or intolerance) are recorded in the case report forms. [b] Started after the procedure for insomnia; not a migraine preventive. [c] Eligibility was confirmed by the treating neurologist at screening; the individual agents are not recorded in the trial database. [d] Not a class listed in the EHF consensus; shown for completeness.

Table S12. Days with acute (symptomatic) medication per 28-day period, by patient

Patient	Baseline	4 wk	12 wk	24 wk	36 wk	48 wk	Acute medication at baseline	ICHD-3 overuse class
1	24	18	9	8	9	8	Tramadol; simple analgesics	Opioid
2	22	20	0	0	0	0	Ibuprofen; paracetamol	Simple analgesic
3	20	21	17	17	15	17	Naproxen; paracetamol	Simple analgesic
4	19	17	0	0	0	0	Ibuprofen; paracetamol	Simple analgesic
5	19	18	20	21	18	17	Naproxen; ibuprofen	Simple analgesic
6	17	16	16	16	19	18	Paracetamol; naproxen	Simple analgesic
7	20	15	0	0	0	0	Ibuprofen; paracetamol	Simple analgesic
8	18	23	20	19	16	18	Ibuprofen; naproxen	Simple analgesic
9	21	20	1	3	0	—	Paracetamol; ibuprofen	Simple analgesic
10	23	16	6	5	6	—	Aspirin; nimesulide; paracetamol–codeine–caffeine combination; sumatriptan	Multiple drug classes

Values are the number of days on which any acute medication was taken within the same 28-day periods as in Table S9; baseline is the 28-day screening period. Em dash denotes a period not yet reached at the data cut-off of 5 September 2026. At baseline all 10 patients exceeded the ICHD-3 frequency thresholds for medication overuse (≥15 days per month for simple analgesics, ≥10 days for opioids, combination analgesics, triptans or multiple classes); the operationalisation of the medication-overuse exclusion criterion is described in Section 2.2.2 (Headache-Specific Exclusions).

Table S13. SPADES grades assigned by the operator and by the second reader, and the consensus grade, for each target branch

Patient	Branch	Operator	Second reader	Consensus (Table 2)
1	Right frontal	III	III	III
	Right parietal	III	III	III
	Left frontal	III+Vens	**III ***	III+Vens
	Left parietal	III+Vens	**III ***	III+Vens
2	Right frontal	III+Vens	III+Vens	III+Vens
	Right parietal	III	III	III
	Left frontal	III	III	III
	Left parietal	III	III	III
3	Right frontal	III	III	III
	Right parietal	0	0	0
	Left frontal	III+Vens	III+Vens	III+Vens
	Left parietal	III	III	III
4	Right frontal	II+Vens	II+Vens	II+Vens
	Right parietal	III	III	III
	Left frontal	IV+Vens	IV+Vens	IV+Vens
	Left parietal	III	III	III
5	Right frontal	II	II	II
	Right parietal	I	I	I
	Left frontal	0	0	0
	Left parietal	0	0	0
6	Right frontal	II	II	II
	Right parietal	II	II	II
	Left frontal	III	III	III
	Left parietal	0	0	0
7	Right frontal	III+Vens	III+Vens	III+Vens
	Right parietal	III	III	III
	Left frontal	IV+Vens	IV+Vens	IV+Vens

Patient	Branch	Operator	Second reader	Consensus (Table 2)
	Left parietal	II	II	II
8	Right frontal	III+Vens	**III ***	III+Vens
	Right parietal	III	III	III
	Left frontal	III+Vens	III+Vens	III+Vens
	Left parietal	II	**III ***	II
9	Right frontal	III	III	III
	Right parietal	III+Vens	III+Vens	III+Vens
	Left frontal	III	III	III
	Left parietal	III+Vens	III+Vens	III+Vens
10	Right frontal	IV+Vens	IV+Vens	IV+Vens
	Right parietal	III	III	III
	Left frontal	III+Vens	III+Vens	III+Vens
	Left parietal	III	III	III

Grades: 0, target branch not embolized; I–IV, arterial penetration level (Section 2.5); +Vens, venous extension. The operator assigned grades at the time of the procedure; the second reader, an interventional neuroradiologist blinded to clinical outcome, re-scored all 40 branches from the intraoperative craniograms and final angiographic runs. Asterisks mark the 4 branches in which the second reader disagreed with the operator (three on venous extension, one on arterial level); in all four the consensus grade corresponded to the operator’s grade. With the second reader’s grades, technical success (grade ≥III) would have been 30 of 40 branches and 5 of 10 patients, versus 29 of 40 and 4 of 10 with the consensus grades.

Table S14. Venous structures reached by the embolic agent in the 14 branches with venous extension (8 patients)

Patient	Branch	SPADES	Meningeal companion veins	Lacunae of Trolard	Sphenoparietal sinus	Superior sagittal sinus	Cortical vein	Diploic venous channels	Injection stopped because of venous spread	Follow-up DSA venous phase
1	Left frontal	III+Vens	Yes	No	Wall	No	No	No	No	Patent
1	Left parietal	III+Vens	Yes	No	No	No	No	Yes (petrosquamosal)	No	Patent
2	Right frontal	III+Vens	Yes	No	No	No	No	No	No	Patent
3	Left frontal	III+Vens	Yes	No	No	No	No	No	No	Patent
4	Right frontal	II+Vens	Yes	No	No	No	No	No	No	Patent
4	Left frontal	IV+Vens	Yes	Yes	No	No	No	No	Yes	Patent
7	Right frontal	III+Vens	Yes	Yes	Wall	No	No	No	Yes	Patent
7	Left frontal	IV+Vens	Yes	No	No	No	No	No	No	Patent
8	Right frontal	III+Vens	Yes	Yes	No	No	No	No	Yes	Not yet due
8	Left frontal	III+Vens	Yes	Yes	No	Wall	No	No	Yes	Not yet due
9	Right parietal	III+Vens	No	No	No	No	No	Yes	No	Not yet due
9	Left parietal	III+Vens	No	No	No	No	No	Yes	No	Not yet due
10	Right frontal	IV+Vens	Yes	Yes	Wall	No	No	No	Yes	Not yet due
10	Left frontal	III+Vens	Yes	No	Wall	No	No	No	Yes	Not yet due
**Total (14 branches)**			**12**	**5**	**4**	**1**	**0**	**3**	**6**	**Patent 8 / not yet due 6**

Venous extension (+Vens) denotes penetration of the embolic agent beyond the arterial bed into the venous drainage of the dura mater: meningeal companion veins of the embolized branch, lacunae of Trolard, the wall of a dural sinus, or diploic venous channels. “Wall” denotes contact of the embolic cast with the wall or edge of the sinus without filling of its lumen; the lumen of a dural sinus and cortical (bridging) veins were not entered in any branch. Venous structures were assessed on the venous phase of the intraoperative angiographic runs; the follow-up column gives the status of the venous structures reached and of the adjacent sinuses on the venous phase of follow-up digital subtraction angiography (Section 3.4), which had not yet fallen due in patients 8–10 at the data cut-off.
